# Fitness Cost Evolution of Natural Plasmids of Staphylococcus aureus

**DOI:** 10.1128/mBio.03094-20

**Published:** 2021-02-23

**Authors:** Pedro Dorado-Morales, M. Pilar Garcillán-Barcia, Iñigo Lasa, Cristina Solano

**Affiliations:** a Laboratory of Microbial Pathogenesis, Navarrabiomed–Universidad Pública de Navarra–Complejo Hospitalario de Navarra, Instituto de Investigacion Sanitaria de Navarra, Pamplona, Navarra, Spain; b Instituto de Biomedicina y Biotecnología de Cantabria, Universidad de Cantabria—Consejo Superior de Investigaciones Científicas, Santander, Cantabria, Spain; Institut Pasteur

**Keywords:** plasmid, fitness cost, evolution, *Staphylococcus aureus*, CRISPR, insertion sequences, antibiotic resistance

## Abstract

Plasmids have largely contributed to the spread of antimicrobial resistance genes among *Staphylococcus* strains. Knowledge about the fitness cost that plasmids confer on clinical staphylococcal isolates and the coevolutionary dynamics that drive plasmid maintenance is still scarce. In this study, we aimed to analyze the initial fitness cost of plasmids in the bacterial pathogen Staphylococcus aureus and the plasmid-host adaptations that occur over time. For that, we first designed a CRISPR (clustered regularly interspaced palindromic repeats)-based tool that enables the removal of native S. aureus plasmids and then transferred three different plasmids isolated from clinical S. aureus strains to the same-background clinical cured strain. One of the plasmids, pUR2940, obtained from a livestock-associated methicillin-resistant S. aureus (LA-MRSA) ST398 strain, imposed a significant fitness cost on both its native and the new host. Experimental evolution in a nonselective medium resulted in a high rate pUR2940 loss and selected for clones with an alleviated fitness cost in which compensatory adaptation occurred via deletion of a 12.8-kb plasmid fragment, contained between two IS*Sau10* insertion sequences and harboring several antimicrobial resistance genes. Overall, our results describe the relevance of plasmid-borne insertion sequences in plasmid rearrangement and maintenance and suggest the potential benefits of reducing the use of antibiotics both in animal and clinical settings for the loss of clinical multidrug resistance plasmids.

## INTRODUCTION

Plasmids are circular extrachromosomal DNA elements capable of semiautonomous replication by recruiting host cell enzymes (1). Plasmids carry genes necessary for plasmid replication and transmission, and the so-called accessory genes that under specific environmental conditions provide beneficial traits, such as antibiotic resistance, tolerance to heavy metals, virulence, metabolism of carbon sources, or root nodulation (2). Many plasmids are large or found in high copy numbers, and logic suggests that in the absence of selection for plasmid-encoded traits, the fitness cost of plasmid carriage should be higher than its benefits and, consequently, clones lacking the plasmid should outcompete the plasmid-bearing population in few generations (3, 4). Some plasmids solve this puzzle by moving the beneficial genes to the bacterial chromosome and then remaining as smaller plasmids or simply disappearing (5). However, this strategy seems not to be the preferred solution, since plasmids containing accessory genes are widely distributed, indicating that mechanisms have been developed to persist in the host, independently of the presence of positive selection. In 2012, a seminal publication by Harrison and Brockhurst proposed that the paradox in plasmid persistence can be explained, at least in part, by compensatory mutations that the plasmid and the host accumulate over time to alleviate the fitness cost (6). Since then, several studies have pursued this line of investigation and found that host-plasmid coevolution is easily achievable in the laboratory through mutations that can occur in the plasmid, the bacterial chromosome, or both (7–10). However, the rules governing plasmid fitness cost remain largely unexplored, and thus, it is not possible to predict the evolution of the same plasmid in different bacterial clones and the fitness effects of different plasmids in the same strain (4).

Staphylococcus aureus is a major human pathogen routinely isolated as a commensal organism in more than a third of the human population. In particular, methicillin-resistant Staphylococcus aureus (MRSA) is a high-priority pathogen according to the World Health Organization list for which new antibiotics are urgently needed. Most natural strains of S. aureus contain different types of mobile genetic elements (MGEs), including plasmids, transposons, insertion sequences, bacteriophages, and pathogenicity islands, that facilitate the acquisition of genes encoding mechanisms of resistance against antimicrobials, biocides, and heavy metals (11). Regarding plasmids, most S. aureus clinical isolates harbor one to three plasmids that can carry antibiotic and antiseptic resistance genes (responsible for resistance to β-lactams, tetracycline, erythromycin, kanamycin, vancomycin, trimethoprim, chloramphenicol, mupirocin, linezolid, teicoplanin, heavy metals, quaternary ammonium compounds, and chlorhexidine), which provide an advantage to the bacterium when the antibiotic and the biocide are present (12). Very often, the same plasmid accumulates multiple antimicrobial resistance genes, which increases the possibilities of selection for plasmid-encoded traits (13). Current knowledge about the fitness cost associated with the carriage of antibiotic resistance plasmids in S. aureus is scarce. The lab of J. Lindsay showed that differences in fitness between two prevalent MRSA clones were unrelated to the presence of large antibiotic resistance plasmids (14). On the other hand, a recent study investigating the coevolution over the last 32 years of the pSK1 plasmid family in the Australian S. aureus ST239 MRSA clade revealed that pSK1 plasmid maintenance is linked to multiple structural variations caused by the insertion sequences IS*256* and IS*257* (15). In summary, we still have a limited understanding of how plasmids and S. aureus have coevolved in order to reduce the fitness cost that plasmid carriage produces when bacteria grow in an antibiotic-free environment, as occurs when patients finish antibiotic treatment. This knowledge could have important consequences in the design of antibiotic management plans, especially if plasmid-bearing bacteria show a lower fitness than plasmid-free bacteria in the absence of antibiotics.

In this work, we addressed the fitness cost and the mechanisms of coevolution of natural plasmids of S. aureus. For that, we generated a CRISPR (clustered regularly interspaced palindromic repeats)-based tool to remove plasmids from natural isolates of S. aureus. One of the resulting plasmid-free strains was transformed with three different plasmids isolated from clinical MRSA strains representative of livestock-associated, hospital, and community environments, and then the initial cost upon entering this new host was calculated and experimental evolution in the absence of selection was assayed. Our results showed that two plasmids did not produce any discernible cost and were stably maintained during evolution. In contrast, a third plasmid entailed a high fitness cost and was rapidly lost in the absence of selective pressure. Adaptation over time was dependent on plasmid rearrangements through plasmid-borne IS*Sau10* insertion sequences and driven by the cost of a plasmid region comprising several antimicrobial resistance genes. These results indicate that insertion sequence-mediated loss of a plasmid region encompassing resistance genes alleviates the cost associated with plasmid carriage in the absence of antibiotics and suggest that limiting antibiotic utilization may select for mutated plasmid-carrying clones that no longer confer resistance.

## RESULTS

### Curing of naturally occurring plasmids of S. aureus using a CRISPR-Cas9 system.

**FIG 1:**
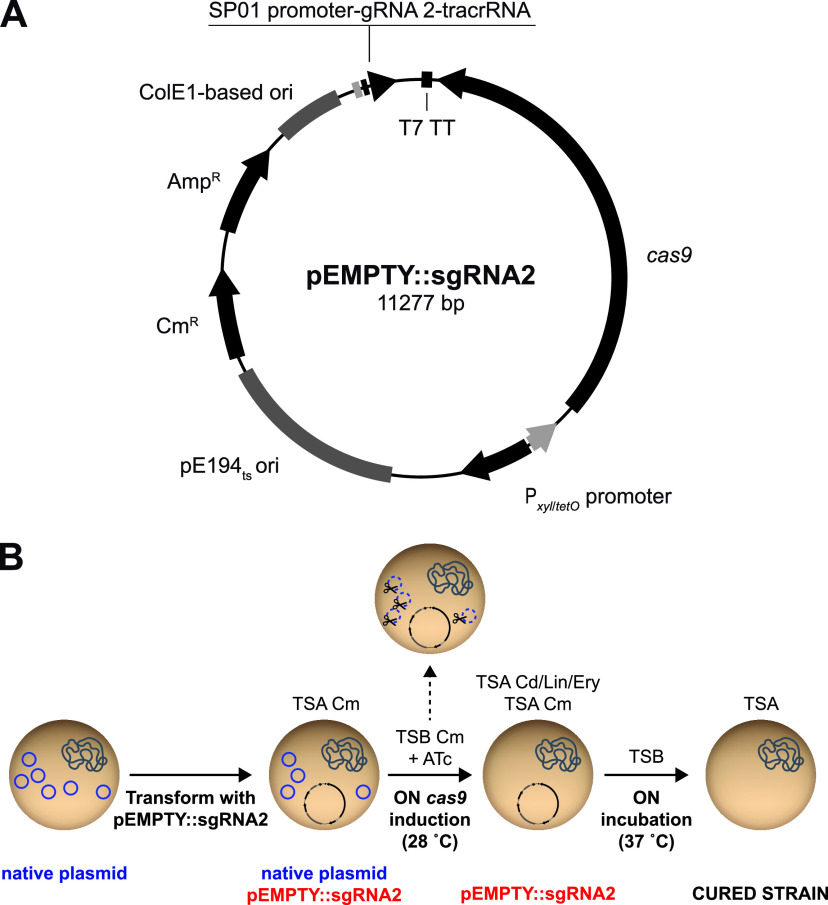
Plasmid map of pEMPTY::sgRNA2 and the plasmid curing process in S. aureus using the pEMPTY::sgRNA2 system. (A) The shuttle vector pEMPTY::sgRNA2 contains the *cas9* gene downstream of the tetracycline-inducible promoter P*_xyl_*_/_*_tetO_* to guarantee regulation of curing functionality, the temperature-sensitive origin of replication pE194_ts_-ori, which allows the loss of the vector system by increasing the temperature to 37°C, and a small guide RNA, consisting of guide RNA 2, which targets S. aureus plasmids fused to the scaffold tracrRNA sequence, downstream of the constitutive SP01 promoter. (B) pEMPTY::sgRNA2 is transformed into a S. aureus strain containing the target plasmid for curing, and transformants are selected on medium containing chloramphenicol as a selective agent. Cells are transferred into liquid TSB medium containing chloramphenicol and anhydrotetracycline (ATc), and the culture is incubated overnight at 28°C to allow *cas9* gene expression and, thus, cleavage of target plasmids directed by guide RNA 2. The culture is plated on TSA containing chloramphenicol and antibiotic (cadmium, lincomycin, or erythromycin) selective TSA, and cured cells are identified by antibiotic sensitivity and PCR screening. A colony of cured cells is incubated overnight in nonselective TSB medium at 37°C, and the culture is plated on nonselective TSA and incubated overnight at 37°C to select a cured colony that has lost plasmid pEMPTY::sgRNA2.

In order to develop an efficient plasmid curing tool for S. aureus, we constructed a plasmid that enables CRISPR-Cas9 precise targeting of S. aureus plasmid DNA. For that, the *cas9* gene was cloned downstream of the tetracycline-inducible promoter P*_xyl_*_/_*_tetO_* inside a pCN38 backbone in which the replication origin was changed by the temperature-sensitive origin of replication pE194_ts_-ori, leading to a thermosensitive plasmid that we named pEMPTY (see Fig. S1 in the supplemental material). Then, to identify guide RNAs that target the majority of natural plasmids found in S. aureus, we searched for fully sequenced plasmids from the genus *Staphylococcus* in the NCBI RefSeq database. Replication origins (*oriT*) and *rep* and *mob* genes, which codify replication initiation proteins and relaxases, respectively, were queried using the online platform sgRNA scorer 2.0 (16) to predict guide RNA activity on input sequences. Only target sequences that were present in 10 or more plasmids with a score equal to or greater than 0.5 were selected (Data Set S1). From these, the sequence targeted by guide RNA 2 (gRNA2) was selected, since it was found in 56% of S. aureus plasmids. Then, gRNA2 was fused to the transactivating RNA (tracrRNA) and cloned under the control of the constitutive strong promoter SP01 in plasmid pEMPTY, leading to the final thermosensitive plasmid curing tool pEMPTY::sgRNA2 (Fig. 1A; also, see Fig. S1).

10.1128/mBio.03094-20.4 FIG S1 Schematic representation of the construction of the pEMPTY::sgRNA2 plasmid. Download FIG S1, PDF file, 0.1 MB. Copyright © 2021 Dorado-Morales et al. 2021 Dorado-Morales et al. https://creativecommons.org/licenses/by/4.0/ This content is distributed under the terms of the Creative Commons Attribution 4.0 International license.

10.1128/mBio.03094-20.8 DATA SET S1 sgRNA site identification and activity on staphylococcal plasmid-related sequences. Download Data Set S1, XLSX file, 0.09 MB. Copyright © 2021 Dorado-Morales et al. 2021 Dorado-Morales et al. https://creativecommons.org/licenses/by/4.0/ This content is distributed under the terms of the Creative Commons Attribution 4.0 International license.

To test the efficiency of our plasmid-curing system, we first transformed S. aureus RN4220 with seven unrelated plasmids of similar size and diverse origins targeted by single guide RNA 2 (sgRNA2) (pMW2, pUR2940, pN315, pLAC-p03, pUR1902, pUR3912, and pUR1841) (Table 1). Also, plasmid pUR2355 was used as a control of plasmid curing unavailability because it does not contain the gRNA2 target DNA sequence. S. aureus RN4220 plasmid-bearing strains were transformed with pEMPTY::sgRNA2, *cas9* expression was induced with anhydrotetracycline, and bacteria were plated on general Trypticase soy agar (TSA) medium and TSA containing a selective agent to calculate curing rates (Fig. 1B). All S. aureus RN4220 derivatives, except for the control strain bearing pUR2355, became sensitive to the selective agent with plasmid curing efficiencies that ranged between 98 and 100% of the total population (Table 2).

**TABLE 1:** Characteristics of plasmids used to test the curing efficiency of pEMPTY::sgRNA2

Plasmid name	Origin	Size (pb)	GenBank no.	gRNA2 target sequence	Resistance determinant(s)	Toxin-antitoxin system	Encoded bacteriocin
pMW2	S. aureus MW2 (CA-MRSA); clonal lineage USA400; CC1 (17)	20654	NC_005011.1	Yes	*cadDX*, *blaIRZ*	Type I, Fst family	Lactococcin
pUR2940	S. aureus C2940 (LA-MRSA); ST398; CC398 (18)	23702	MW367469	Yes	*cadDX*, *erm*(C), *erm*(T), *dfrK*, *tet*(L), *copA*	None	None
pN315	S. aureus N315 (HA-MRSA); clonal lineage USA100; CC5 (19)	24653	NC_003140.1	Yes	*cadDX*, *blaIRZ*, *arsBC*	Type I, Fst family	Lactococcin
pLAC-p03	S. aureus LAC (CA-MRSA); clonal lineage USA300; CC8 (20, 50)	27068	NZ_CP002149.1	Yes	*cadDX*, *msrA*, *mphC*, *blaIRZ*, *aph*(3′)-IIIa	Type I, Fst family	None
pUR1902	S. aureus C1902 (LA-MRSA); ST398; CC398 (18)	≈2000	HF583291.1 (partial sequence)	Yes	*copA*, *mco*, *tet*(L), *cadDX*, *erm*(T), *aadD*	None in the available sequence	None in the available sequence
pUR3912	S. aureus C3912 (LA-MSSA); ST398; CC398 (51, 52)	6176	HE805623.2	Yes	*cadDX*, *erm*(T)	None	None
pUR1841 (pLNU1)	S. aureus C1841 (LA-MRSA); clonal lineage ST398; CC398 (53)	2361	AM184099.1	Yes	*lnu*(A)	None	None
pUR2355	S. aureus C2355 (LA-MRSA); clonal lineage ST398; CC398 (54)	7609	JQ312422.1	No	*vga*(A)	None	None

**TABLE 2:** Plasmid curing efficiencies achieved with the use of pEMPTY::sgRNA2

Strain	Curing efficiency (%)
RN4220 pMW2	99.9
RN4220 pUR2940	99.7
RN4220 pN315	100
RN4220 pLAC-p03	100
RN4220 pUR1902	99.9
RN4220 pUR3912	98.3
RN4220 pUR1841	99.9
RN4220 pUR2355^*a*^	0
MW2	100
C2940	96.1
N315	98
LAC	96.1
C1902	100
C3912^*b*^	Killed
C1841	100
C2355^*a*^	0

a Plasmid pUR2355 does not contain the guide RNA 2 target DNA sequence.

b Plasmid pUR3912 was present as an extrachromosomal element as well as integrated into the chromosome of strain C3912 (52).

Next, we evaluated the efficacy of pEMPTY::sgRNA2 for curing plasmids in their original wild-type strains where transformation efficiencies can be compromised. In all the strains, our system showed plasmid curing rates similar to those previously obtained in the laboratory strain RN4220 (Table 2). To ensure that curing efficiencies shown in Table 2 were due to the CRISPR-Cas9 activity of pEMPTY::sgRNA2, the pEMPTY plasmid, which does not carry any sgRNA, was used in parallel. In this case, plasmid maintenance at the end of the curing experiment was 100% in all cases (data not shown).

Altogether, these results demonstrate that pEMPTY::sgRNA2 can be efficiently used for curing naturally occurring plasmids from S. aureus isolates of diverse origins. The replacement of sgRNA2 by a different guide RNA would allow the removal of plasmids that are not targeted by sgRNA2.

### Evaluation of the fitness cost and evolutionary adaptation associated with plasmid carriage in S. aureus.

**FIG 2:**
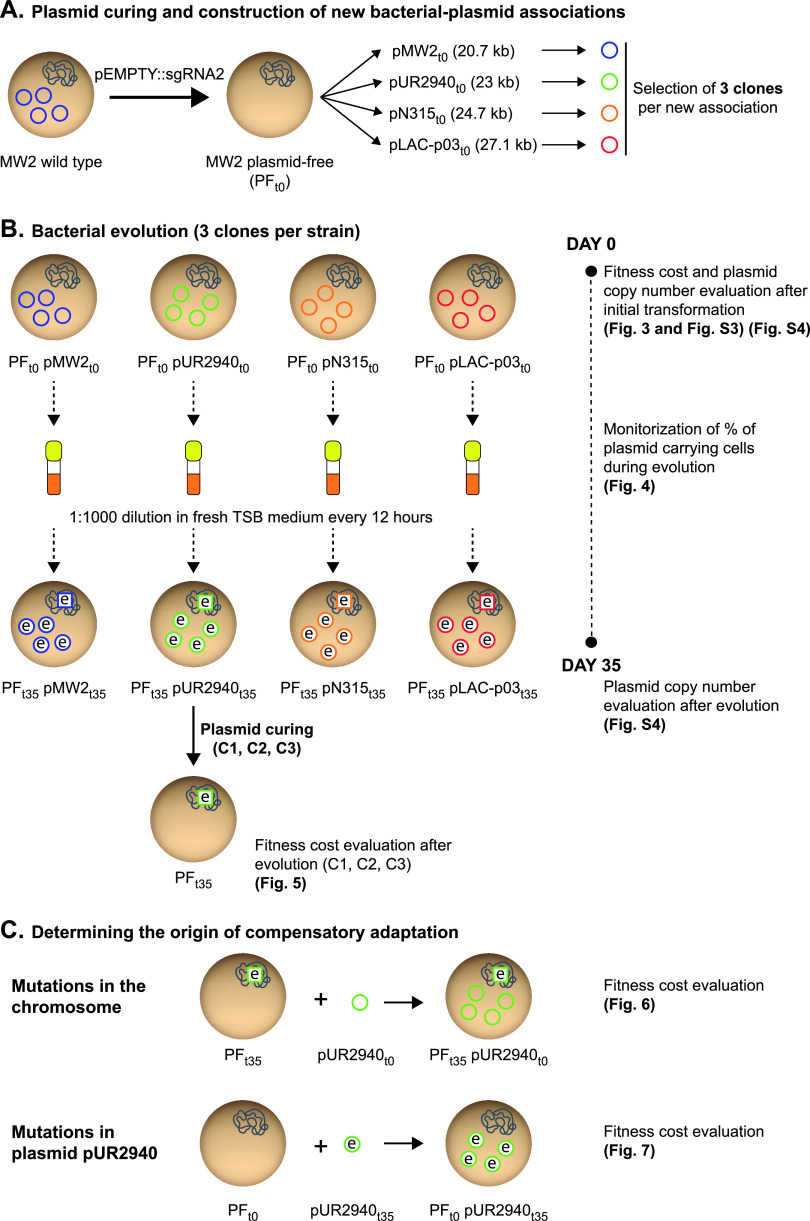
Experimental design of the evolution process of S. aureus MW2 plasmid-containing clones and analysis of the compensatory adaptation to plasmid pUR2940. Bacterial cells are represented by brown circles and include chromosomes depicted in blue. Plasmids are indicated by colored circles. The letter “e” indicates either an evolved strain or evolved plasmid.

**FIG 3:**
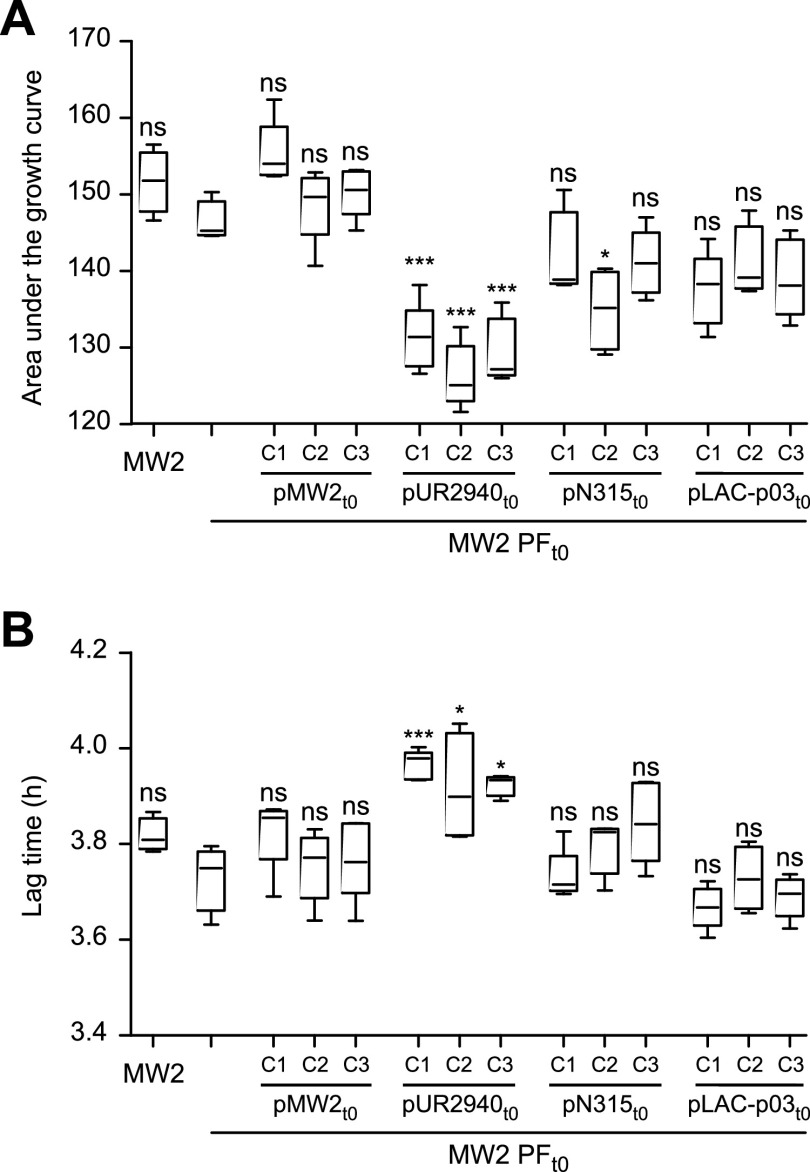
Initial fitness cost after transformation of plasmid-free MW2 strain with plasmids pMW2, pUR2940, pN315, and pLAC-p03. The fitness of plasmid-free MW2 (MW2 PF_t0_) was compared with that of the wild-type MW2 strain and MW2 PF_t0_ transformed with plasmids pMW2, pUR2940, pN315, and pLAC-p03 (three independently transformed clones [C1, C2, and C3] were analyzed in each case). (A) Area under the growth curve; (B) duration of the lag phase. Data were collected during growth in TSB medium for 24 h at 37°C with shaking. Ten technical replicates were used for each of the tested clones. Results from one representative experiment of at least three independent experiments are shown. Plasmid pUR2940 produced a significant cost in cured strain MW2. Statistical analysis was carried out using a one-way analysis of variance combined with the Bonferroni multiple *post hoc* test. *, *P* < 0.05; ***, *P* < 0.001; ns, no significant difference.

The pEMPTY::sgRNA2 plasmid-curing tool enables the study of the fitness of clones with and without a particular plasmid, and also, it permits construction of new bacterium-plasmid associations. Thus, aiming to investigate the fitness cost produced by different plasmids in a cured S. aureus strain, we transformed the plasmid-free derivative of S. aureus MW2 strain (MW2 PF_t0_) with plasmids pUR2940, pN315, and pLAC-p03 (Fig. 2A and Fig. S2). S. aureus MW2 was selected as the recipient strain because of its major significance as a prototype community-associated methicillin-resistant S. aureus (CA-MRSA) strain of the clonal complex 1 (CC1)/USA400 lineage (17). On the other hand, the above-mentioned plasmids (Table 1) were used because they are carried by MRSA strains representative of the livestock-associated CC398 lineage (LA-MRSA C2940 strain) (18), health care-associated CC5/USA100 lineage (HA-MRSA N315 strain) (19), and community-associated CC8/USA300 lineage (CA-MRSA LAC strain) (20) (Table S1). Also, MW2 PF_t0_ was transformed with its own original pMW2 plasmid as a control for neutral fitness cost (Fig. 2A and Fig. S2). Three plasmid-carrying clones selected from three independent transformation rounds were studied in each case (Fig. 2B). Analysis of the initial fitness cost, measured as the differences in both the area under the growth curve and the duration of the lag phase between MW2 PF_t0_ and plasmid-bearing clones, revealed that only plasmid pUR2940 caused a significant decrease in the area under the growth curve and also a delay in the lag phase (Fig. 3 and Fig. S3).

10.1128/mBio.03094-20.1 TABLE S1 Strains used in this study. Download Table S1, DOCX file, 0.04 MB. Copyright © 2021 Dorado-Morales et al. 2021 Dorado-Morales et al. https://creativecommons.org/licenses/by/4.0/ This content is distributed under the terms of the Creative Commons Attribution 4.0 International license.

10.1128/mBio.03094-20.5 FIG S2 Maps of plasmids pMW2, pUR2940, pN315, and pLAC-p03. Images were generated with BLAST Ring Image Generator (BRIG) using plasmid sequences retrieved from the NCBI RefSeq database or obtained by Illumina sequencing. Download FIG S2, PDF file, 0.6 MB. Copyright © 2021 Dorado-Morales et al. 2021 Dorado-Morales et al. https://creativecommons.org/licenses/by/4.0/ This content is distributed under the terms of the Creative Commons Attribution 4.0 International license.

10.1128/mBio.03094-20.6 FIG S3 Growth curve of plasmid-free MW2 (MW2 PF_t0_) compared with that of the wild-type MW2 strain and MW2 PF_t0_ transformed with plasmids pMW2, pUR2940, pN315, and pLAC-p03. Data were collected every 15 min during growth in TSB medium in 96 multiwell plates for 6.5 h at 37°C with shaking. The average OD_595_ values and standard deviations for 10 technical replicates were plotted for each of the tested strains. In the case of MW2 PF_t0_ transformed with plasmids pMW2, pUR2940, pN315, and pLAC-p03, results for one representative clone of the three independently transformed clones are shown. Download FIG S3, PDF file, 0.1 MB. Copyright © 2021 Dorado-Morales et al. 2021 Dorado-Morales et al. https://creativecommons.org/licenses/by/4.0/ This content is distributed under the terms of the Creative Commons Attribution 4.0 International license.

**FIG 4:**
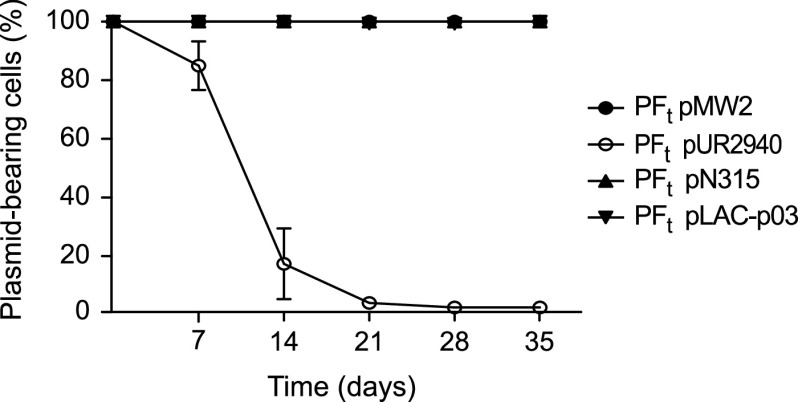
Plasmid stability in the bacterial population during the evolution process. The proportion of plasmid-bearing bacteria is shown as a function of the evolution time. Every 7 days during the evolution process, cultures of the three clones corresponding to each MW2 PF strain transformed with plasmids pMW2, pUR2940, pN315, and pLAC-p03 were plated on TSA medium. One hundred colonies from each culture were streaked on TSA and TSA containing 0.05 mM Cd as a plasmid-selective agent. The numbers of total and resistant colonies were determined, and the percent plasmid-carrying colonies was calculated. Data are means and standard deviations of values obtained from the three individual clones analyzed in each case.

In order to analyze the evolution of newly acquired plasmids over time in the absence of selection, we propagated the MW2 plasmid-carrying strains in antibiotic-free culture medium for 35 days, diluting the culture (1:1,000) every 12 h, which corresponds to approximately 640 generations of bacterial evolution by the end of the experiment (Fig. 2B). Evaluation of the dynamics of plasmid carriage over the 35 days of the experiment showed that while the control plasmid pMW2 and also plasmids pN315 and pLAC-p03 were stably maintained by the entire population, the frequency of pUR2940 plasmid-carrying cells rapidly declined, with almost 100% of the population of all three evolved clones being plasmid free by the end of the assay (Fig. 4). At day 35, cultures were plated on TSA medium containing 0.05 mM cadmium, and one plasmid-bearing clone was isolated from each culture. Analysis of the plasmid copy number in all MW2 plasmid-bearing clones at the beginning of the evolution process and also in isolated plasmid-carrying clones at the end of the experiment showed that plasmid copy number did not change during bacterial evolution in all cases (Fig. S4). Together, these results showed that, from the three plasmids analyzed, pUR2940 produced an evident cost in MW2 strain that contributed to a decline of the plasmid frequency in the population over time.

10.1128/mBio.03094-20.7 FIG S4 Plasmid copy number at days 0 and day 35 of the evolution experiment. Numbers of pMW2, pUR2940, pN315, and pLAC-p03 copies per cell in the three ancestral MW2 transformed clones (t0) and the three MW2 plasmid-carrying evolved clones (t35) were determined by qPCR. Data are means and standard deviations of values obtained from the three individual clones analyzed in each case. Experiments were carried out in triplicate. Statistical analysis was carried out using a Mann-Whitney *U* test. ns, no significant difference. Download FIG S4, PDF file, 0.09 MB. Copyright © 2021 Dorado-Morales et al. 2021 Dorado-Morales et al. https://creativecommons.org/licenses/by/4.0/ This content is distributed under the terms of the Creative Commons Attribution 4.0 International license.

**FIG 5:**
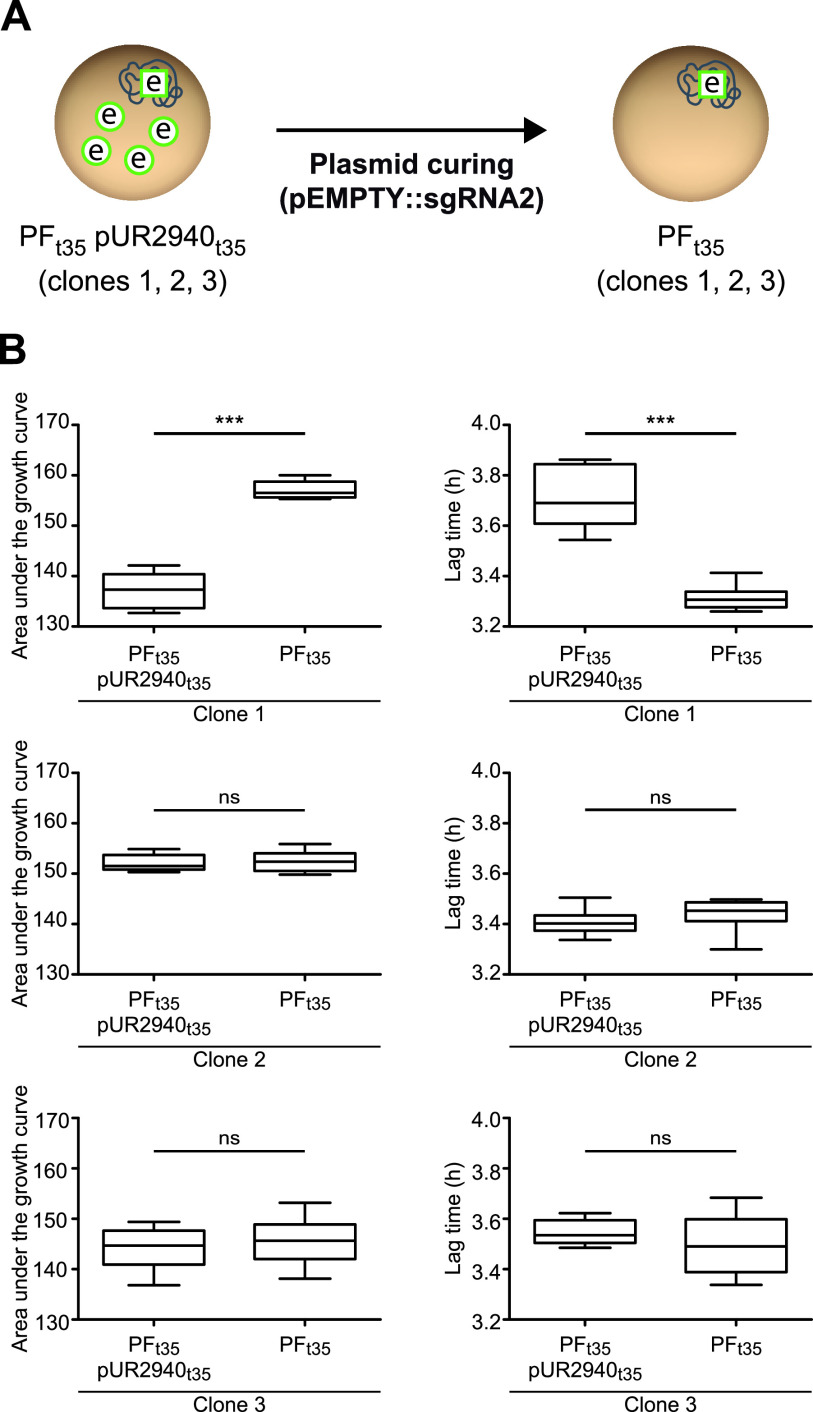
Final fitness cost of plasmid pUR2940 in strain MW2 after the evolution process. (A) To elucidate if plasmid pUR2940 still imposed a cost in the evolved populations, the three MW2 pUR2940 evolved clones (MW2 PF_t35_ pUR2940_t35_ C1, C2, and C3) were cured, leading to their respective cured clones (MW2 PF_t35_ C1, C2, and C3). The fitness of all clones was compared. (B) Area under the growth curve (left) and duration of the lag phase (right). Data were collected during growth in TSB medium for 24 h at 37°C with shaking. Ten technical replicates were used for each of the tested strains. Results from one representative experiment of at least three independent experiments are shown. Statistical analysis was carried out using the Mann-Whitney *U* test. ***, *P* < 0.001; ns, no significant difference.

Next, to discern whether the pUR2940 plasmid still produced a cost in the small population that carried the plasmid at the end of the evolution process or whether compensatory adaptation had taken place, pUR2940 was cured in MW2 pUR2940 evolved clones (MW2 PF_t35_ pUR2940_t35_) and the fitness of evolved plasmid-bearing clones was compared to that of their respective cured clones (MW2 PF_t35_) (Fig. 2B and Fig. 5A). Results showed that pUR2940 no longer produced a cost in two of the three clones analyzed, indicating that compensatory adaptation had occurred during evolution to overcome the cost associated with pUR2940 carriage (Fig. 5B).

### Elucidation of the genetic basis of compensation of the cost produced by plasmid pUR2940.

**FIG 6:**
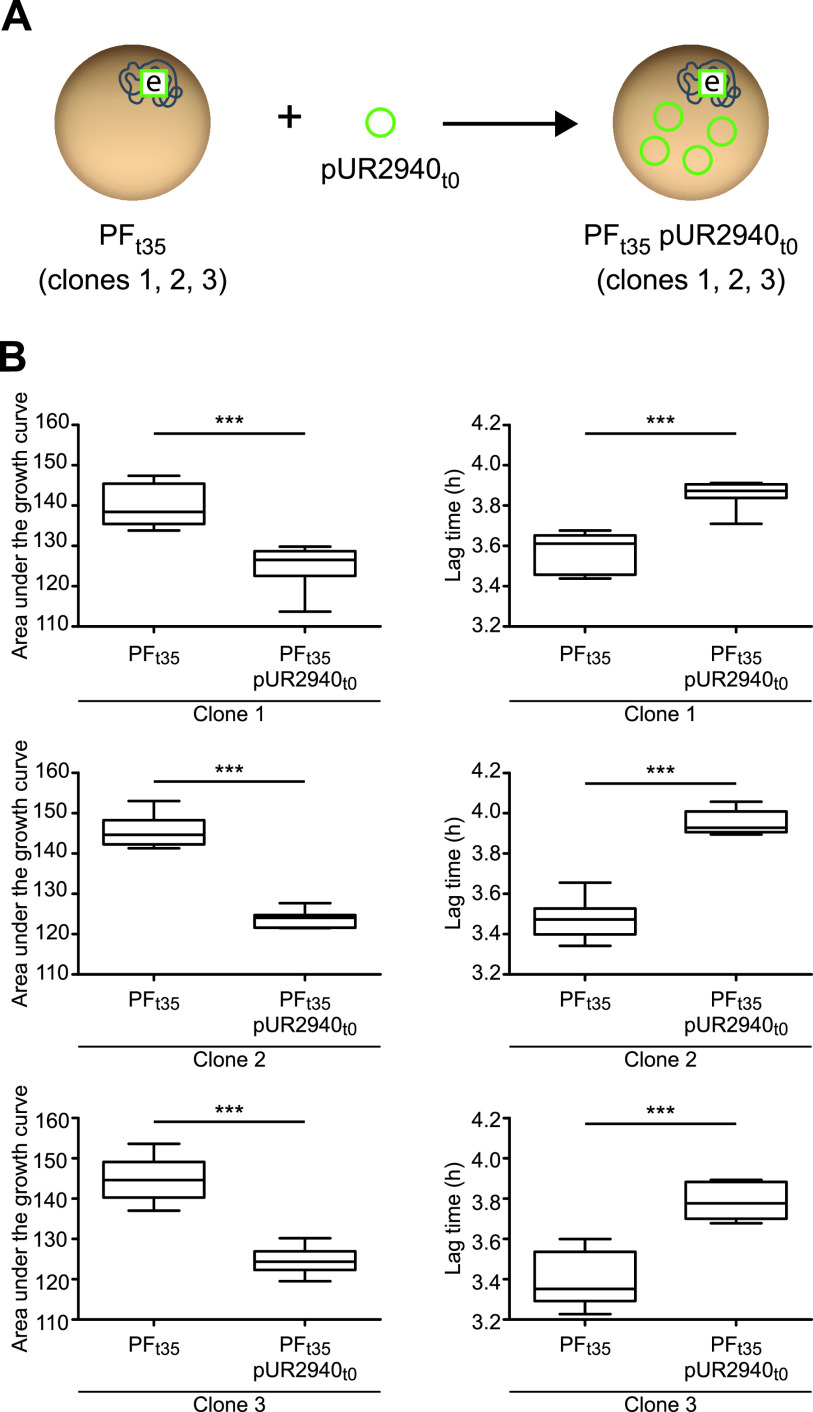
Compensatory adaptation to pUR2940 is not based on changes in the chromosome of host MW2 bacteria. (A) The first strategy to analyze the origin of MW2 compensatory adaptation to pUR2940. Evolved cured clones MW2 PF_t35_ C1, C2, and C3 were transformed with the ancestral pUR2940_t0_ plasmid. (B) The fitness of MW2 PF_t35_ C1, C2, and C3 was compared with that of transformed clones with ancestral pUR2940_t0_. The area under the growth curve (left) and the duration of the lag phase (right) are shown. Data were collected during growth in TSB medium for 24 h at 37°C with shaking. Ten technical replicates were used for each of the tested strains. Results from one representative experiment of at least three independent experiments are shown. Statistical analysis was carried out using the Mann-Whitney *U* test. ***, *P* < 0.001.

**FIG 7:**
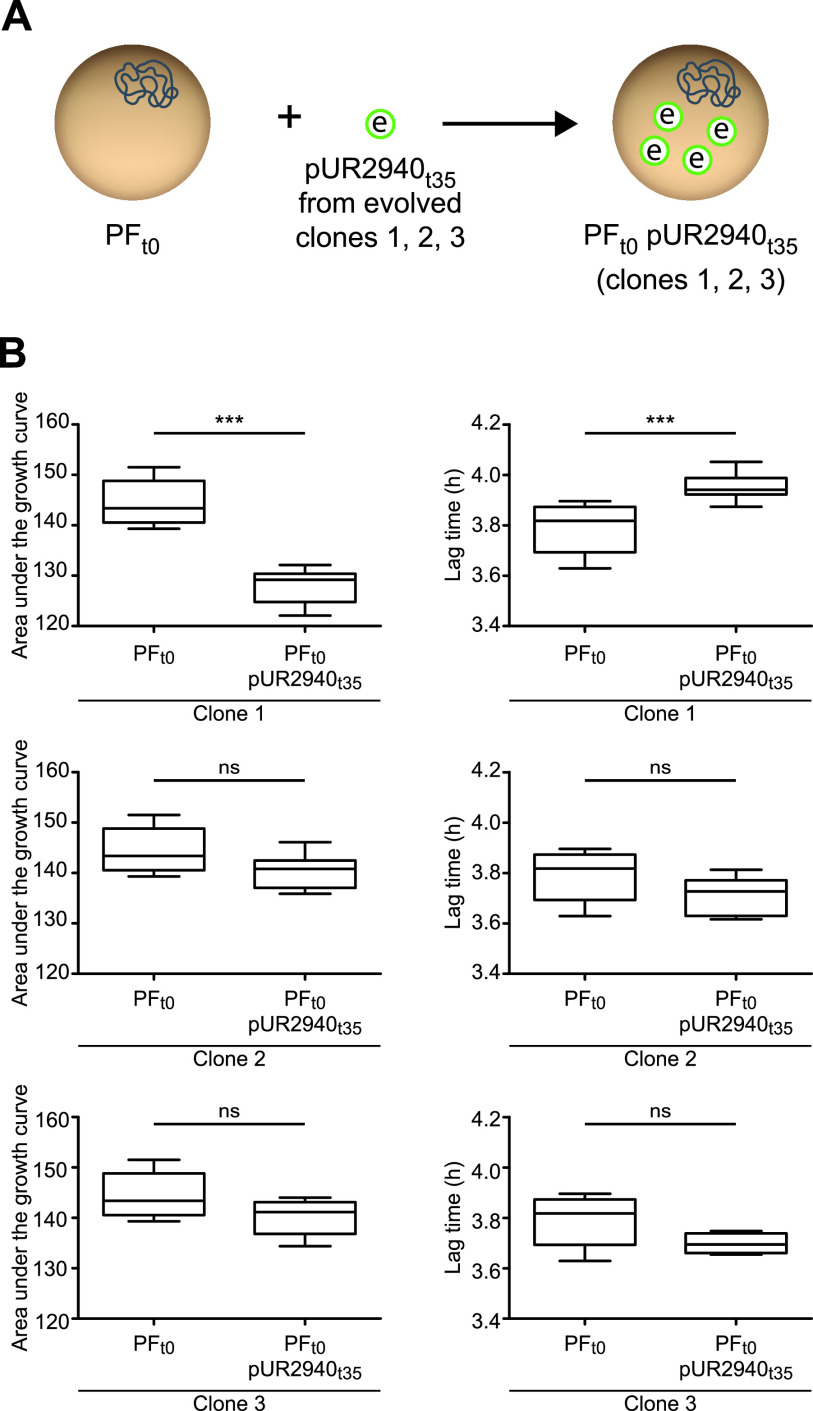
Compensatory adaptation of MW2 to pUR2940 in clones 2 and 3 is due to changes in pUR2940. (A) The second strategy to analyze the origin of MW2 compensatory adaptation to pUR2940. The ancestral MW2 plasmid-free strain, MW2 PF_t0_, was transformed with pUR2940_t35_ plasmids isolated from the three evolved MW2 PF_t35_ pUR2940_t35_ clones. (B) Fitness of ancestral MW2 PF_t0_ was compared before and after transformation with pUR2940_t35_ from evolved clones. The area under the growth curve (left) and the duration of the lag phase (right) are shown. Data were collected during growth in TSB medium for 24 h at 37°C with shaking. Ten technical replicates were used for each of the tested strains. Results from one representative experiment of at least three independent experiments are shown. Statistical analysis was carried out using the Mann-Whitney *U* test. ***, *P* < 0.001; ns, no significant difference.

To gain insight into the genetic basis of MW2 compensatory adaptation to plasmid pUR2940 present in evolved MW2 PF_t35_ pUR2940_t35_ clones, two strategies were followed (21) (Fig. 2C). First, to analyze the contribution of chromosomal modifications to plasmid cost alleviation, we transformed the evolved cured clones (MW2 PF_t35_) with the ancestral pUR2940_t0_ plasmid, and fitness was compared prior to and after transformation with the plasmid (Fig. 6A). All three evolved, cured clones transformed with ancestral pUR2940_t0_ showed low fitness compared to their nontransformed counterparts (i.e., they showed a clear decrease in the area under the growth curve and a delay in the lag phase) (Fig. 6B). These results suggested that chromosomal modifications were not involved in pUR2940 carriage adaptation. Second, to investigate the contribution of pUR2940 plasmid mutations to cost alleviation, we transformed the ancestral MW2 plasmid-free strain, MW2 PF_t0_, with the different pUR2940_t35_ plasmids isolated from the three evolved MW2 PF_t35_ pUR2940_t35_ clones (Fig. 7A). pUR2940 isolated from the evolved adapted MW2 PF_t35_ pUR2940_t35_ clones 2 and 3 did not produce a cost when transformed into the ancestral MW2 plasmid-free strain (Fig. 7B). In contrast, the pUR2940 plasmid variant producing a fitness cost in the evolved MW2 PF_t35_ pUR2940_t35_ clone 1 also produced a cost when it was transformed into the ancestral MW2 plasmid-free strain. Altogether, these results suggested that compensatory adaptation to plasmid pUR2940 present in evolved MW2 PF_t35_ pUR2940_t35_ can occur through mutations in plasmid pUR2940 during the evolution process.

**FIG 8:**
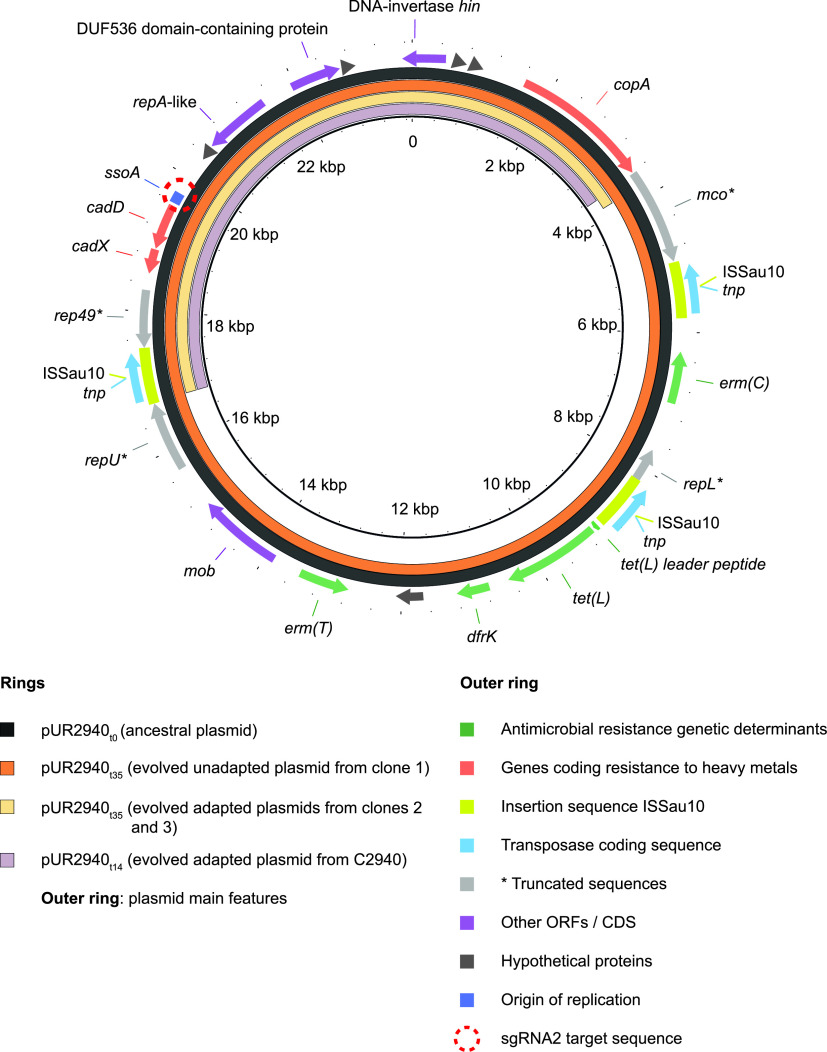
Comparison of the ancestral plasmid pUR2940 and its evolved variants. The ancestral plasmid pUR2940_t0_, evolved plasmid pUR2940_t35_ from MW2 evolved clones 1, 2, and 3, and evolved plasmid pUR2940_t14_ isolated from evolved strain C2940 are compared. Image visualization was carried out with BLAST Ring Image Generator (BRIG), which shows the similarity between the central reference sequence pUR2940_t0_ and the other variants as concentric rings. ORFs, open reading frames; CDS, coding sequence.

To investigate the changes in pUR2940_t35_ responsible for the adaptation, we sequenced the pUR2940_t35_ variants isolated from the two evolved adapted clones and compared the retrieved sequences to that of pUR2940_t35_ isolated from the nonadapted clone and also to the ancestral plasmid pUR2940_t0_. As predicted by the fitness experiments, no mutations were found in the plasmid from the nonadapted clone while a fragment of 12,880 bp was lost in both plasmid variants isolated from adapted clones (Fig. 8). Its loss seemed to be the result of homologous recombination involving two copies of the insertion sequence IS*Sau10.* This fragment was not found as an independent replicative unit (it includes replication initiation protein genes, although most likely incomplete), nor was it inserted into the host chromosome of the evolved strain. Notably, the lost fragment contains genes that provide resistance to macrolides, lincosamides, and streptogramin B (*ermC* and *ermT*), trimethoprim (*dfrK*), and tetracycline (*tetL*). This fragment also includes the MOB_V_ relaxase gene and thus, the conjugative mobilization of these evolved plasmid variants would be impaired.

Finally, in order to confirm that this new plasmid variant was indeed completely stable in the MW2 strain, we propagated the ancestral strain MW2 PF_t0_ transformed with pUR2940_t35_ evolved plasmids for 14 days, which corresponds to approximately 256 generations of bacterial evolution, following the previously described evolution process (Fig. 2B). After 14 days of evolution, the percentage of plasmid carriage was calculated, and as expected, the two new adapted variants pUR2940_t35_ were present in 100% of the population whereas the nonadapted plasmid pUR2940_t35_ was almost lost (4% of plasmid-bearing bacteria). Overall, these results demonstrated that pUR2940 plasmid reorganization is sufficient to alleviate the cost produced in the MW2 strain.

### pUR2940 also imposes a cost in its natural host that can be compensated for via IS*Sau10*-mediated deletion of a costly region from the plasmid.

**FIG 9:**
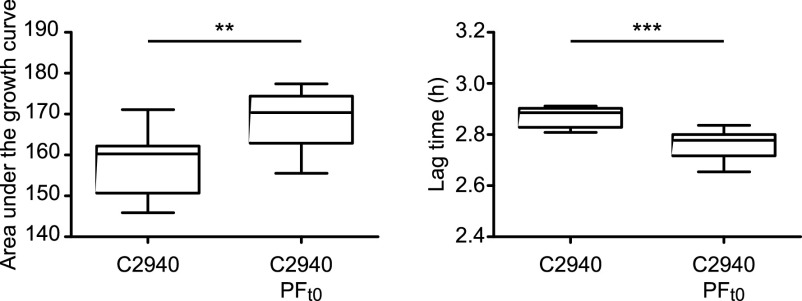
Fitness cost of plasmid pUR2940 in its original host, strain C2940. Fitness of strain C2940 was compared with that of its plasmid-free derivative C2940 PF_t0_. (Left) Area under the growth curve; (right) duration of the lag phase. Data were collected during growth in TSB medium for 24 h at 37°C with shaking. Ten technical replicates were used for each of the tested strains. Results from one representative experiment of at least three independent experiments are shown. Statistical analysis was carried out using the Mann-Whitney *U* test. **, *P* < 0.01; ***, *P* < 0.001.

The above results indicated that the pUR2940 plasmid produces a noticeable cost in the MW2 strain that can be alleviated through plasmid rearrangements when cultured in the absence of antibiotic pressure. Based on this, we wondered whether pUR2940 also imposes a cost in the original S. aureus strain (C2940) under conditions in which plasmid genes do not provide any benefit to the host. A comparison of the fitness of the C2940 strain and its respective cured derivative, when grown in the absence of selection, confirmed that pUR2940 produces a significant cost in its original host (Fig. 9). Indeed, when C2940 was propagated in liquid medium without antibiotics for 14 days, only 4.6% of the cells retained the ancestral plasmid. Given that the C2940 strain shows an intrinsic high resistance to cadmium (18), we were unable to select cadmium-resistant and erythromycin-sensitive clones, which would presumably harbor an evolved pUR2940 plasmid. To analyze if reorganized pUR2940 variants appear during C2940 evolution in the absence of selection, a plasmid extraction was performed from the whole evolved culture after 14 days of propagation in Trypticase soy broth (TSB) medium, and the PCR product of primers 412 and 414 was examined through agarose gel electrophoresis. A band with the size of the expected evolved pUR2940 plasmid was excised and analyzed by sequencing, which yielded data showing deletion of a fragment similar to the one occurring in pUR2940_t35_ isolated from the MW2 evolved adapted clones (Fig. 8). Hence, these results confirmed the occurrence of pUR2940 reorganization via the IS*Sau10* insertion sequence that leads to the loss of antimicrobial resistance genes when the C2940 strain encounters an antibiotic-free environment.

## DISCUSSION

Location of genes in plasmids rather than in the chromosome offers benefits to bacteria because it provides higher levels of gene expression, different versions of a gene that coexist in the same cell (heterozygosity) and an increase in the mutational supply and hence the chance of acquiring mutations and because it facilitates the exchange of genes and the acquisition of new capacities (22). However, the presence of a plasmid also imposes a fitness cost, and therefore, the fact that plasmids are so abundant in bacteria living in competitive environments when the selection pressure has disappeared, for instance, a patient after completion of antibiotic treatment, seems counterintuitive. Most of our current understanding about plasmid fitness and evolution has been obtained from the study of *Gammaproteobacteria*, and it is important to examine whether similar rules govern plasmid evolution in other clinically relevant bacterial species (7, 8, 10, 23, 24). In this study, we investigated the fitness cost associated with plasmid carriage in clinical isolates of S. aureus, where these mobile genetic elements are major agents in the dissemination of multidrug resistance determinants.

The best method to demonstrate the relationship between the presence of the plasmid and fitness cost is the elimination of the plasmid (curing) from the bacterial population. The cost should disappear in cured derivatives and reappear when the plasmid is reintroduced into the cured strain. Plasmid curing has been traditionally performed by prolonged bacterial growth in the presence of chemical and physical agents, some of which introduce mutations in the DNA or interfere specifically with its replication (25). The risk of these strategies is that they favor the accumulation of mutations, something that is incompatible with investigating the molecular basis of plasmid fitness cost. We addressed this methodological limitation by generating a CRISPR-based tool (the pEMPTY plasmid) that requires only the transformation of a given strain to remove the existing plasmid. In the present study, we used a guide RNA that targets 56% of the currently known plasmids in S. aureus. An easy way to expand the chance of plasmid targeting would be to include additional sgRNA in the pEMPTY plasmid.

We used a clinical S. aureus strain from which we removed its native plasmid (plasmid-free S. aureus MW2) to simulate the scenario of the physical arrival of a plasmid into a new host. The analysis of the fitness cost produced upon arrival revealed somewhat surprising results showing that two plasmids (pN315 and pLAC-p03) did not entail any noticeable cost. These two plasmids are slightly larger than the native MW2 plasmid and contain a different set of antimicrobial resistance genes (Table 1). A reasonable explanation for these results is that during the adaptive evolution of S. aureus MW2 to its native plasmid, bacteria accumulated chromosomal changes to alleviate the cost associated with plasmid carriage, and thus, the introduction of a new plasmid does not require additional coevolution steps. In addition, pMW2, pN315, and pLAC-p03 encode toxin-antitoxin systems that might prevent plasmid loss even in the absence of selective pressure, while pN315 and pMW2 encode bacteriocins that might provide a competitive advantage to the plasmid-bearing cells (Table 1). In contrast, plasmid pUR2940 entailed a high fitness cost both to its native host and the new S. aureus MW2 PF_t0_ strain and was very unstable. pUR2940 is a multiresistant plasmid isolated from a human methicillin-resistant S. aureus strain of the clonal lineage ST398 that carries several antibiotic resistance genes and three copies of the IS*Sau10* insertion sequence (18). Importantly, neither toxin-antitoxin systems nor bacteriocins are encoded in pUR2940 (Table 1). Coevolution of the plasmid and the host through serial passages in an antibiotic-free medium selected for plasmids that had lost a 12.8-kb plasmid region that is flanked by two IS*Sau10* elements and comprises all the antibiotic resistance genes. The sequencing results suggest that deletion occurs via intramolecular recombination between two IS*Sau10* insertion sequences. IS*Sau10* belongs to the IS*6*/IS*26* family (26), whose members are involved in the mobilization of antibiotic resistance genes in Gram-negative bacteria (23, 27). Thus, alleviation of the plasmid fitness cost comes at the expense of losing all the antibiotic resistance determinants. Notably, the deleted region was neither found as an independent replicon nor subsequently integrated into the host chromosome, ruling out the risky outcome of antimicrobial gene fixation.

A question raised by these results is how the deleted region entails such a high fitness cost for the host. It is unsurprising that antibiotic resistance genes provide a selective advantage in the presence of antibiotics, but it is less clear why their presence can be so costly with little or no selective pressure. One possibility is that these genes are expressed at very high levels so that transcription, translation, and/or the subsequent interactions between the proteins responsible for antibiotic resistance and cellular networks affect bacterial physiology. Previous work in Escherichia coli showing that expression of a plasmid-encoded tetracycline efflux pump is highly costly supports this notion (28, 29). Alternatively, the transposase encoded by the IS may interact with other mobile genetic elements carried in the bacterial genome and produce a reduction in bacterial fitness (3). The deleted fragment in the evolved plasmid variant included, besides antibiotic resistance genes and IS*Sau10* copies, a Rep_1 rolling-circle replication (RCR) initiation protein gene (most likely truncated in its 3′-terminal region by an IS*Sau10* copy) and a MOB_V_ relaxase. These plasmid backbone genes might also be responsible for plasmid cost. In fact, plasmid-host adaptations have previously been shown to occur via mutations in the plasmid replication machinery (30) and in chromosomal genes interacting with replication proteins (31). Future research involving targeted deletions from the pUR2940 plasmid will be conducted in order to identify exactly what gene(s) is responsible for plasmid cost.

The resulting new evolved plasmid showed no detectable fitness cost, at least with the resolution of the growth measurements used in our study. This plasmid, in the future, might provide a backbone for the entry of a new carriage. The IS*Sau10* copy that remains in the evolved plasmid variants could be a platform to promote cointegration of another molecule also containing an IS*Sau10* copy via the conservative transposition route or homologous recombination.

In-depth studies carried out in other bacteria have shown that adaptive evolution in conditions selecting for plasmid carriage can increase plasmid stability by diminishing the fitness cost of an initially costly plasmid-host association (7, 10, 23, 32, 33). Thus, periods of selection may provide sufficient time for plasmid-host adaptations to occur, enhancing plasmid persistence once selection is removed (7). Taking this into account, our results showing that plasmid pUR2940 imposes a fitness cost in its native S. aureus strain might indicate that long-term coevolution between the host and the plasmid has not yet occurred. On the other hand, an increase in plasmid persistence as a result of positive selection may not always be the case. It has been demonstrated that antibiotic selection can lead to increased plasmid heterogeneity in E. coli and maintenance of all plasmid-carrying hosts regardless of plasmid stability. Thus, constant selection could interfere with the emergence of stable plasmid variants and thus hinder long-term plasmid persistence (34). With regard to the impact of selective pressures on pUR2940 maintenance in health care settings, further evolution analysis in the presence of antibiotics will be necessary to investigate the molecular basis of pUR2940 host adaptation during positive selection.

Finally, future investigations using *in vivo* animal models are necessary to confirm that insertion sequences mediated rearrangement is a general strategy to reduce the fitness cost of multidrug plasmid carriage in the absence of antibiotics. This knowledge is critical to predict the evolution of plasmids containing insertion sequence elements together with multidrug-resistant genes and may have a significant impact on the choice of antibiotic regimens in order to decrease the probability of plasmid persistence.

## MATERIALS AND METHODS

### Bacterial strains, oligonucleotides, and culture conditions.

Bacterial strains, plasmids, and oligonucleotides used in this work are listed in Tables S1, S2, and S3, respectively. Escherichia coli strains were grown in Luria-Bertani medium (LB; Conda-Pronadisa) at 37°C. S. aureus strains were routinely incubated in Trypticase soy broth (TSB; Conda-Pronadisa) at 37°C. When required, media were supplemented with appropriate antibiotics at the following concentrations: ampicillin (Amp), 100 μg ml^−1^; chloramphenicol (Cm), 20 μg ml^−1^; cadmium (Cd), 0.05 mM; erythromycin (Ery), 10 μg ml^−1^; and lincomycin (Lin), 8 μg ml^−1^. Bacteriological agar (VWR) was used as a gelling agent. A stock solution of 10 mg ml^−1^ anhydrotetracycline (Cayman Chemical Company) in dimethyl sulfoxide (DMSO) was prepared and added to cultures at 100 ng ml^−1^ to induce the expression of *cas9* (pEMPTY and derivatives).

10.1128/mBio.03094-20.2 TABLE S2 Plasmids used in this study. Download Table S2, DOCX file, 0.02 MB. Copyright © 2021 Dorado-Morales et al. 2021 Dorado-Morales et al. https://creativecommons.org/licenses/by/4.0/ This content is distributed under the terms of the Creative Commons Attribution 4.0 International license.

10.1128/mBio.03094-20.3 TABLE S3 Oligonucleotides used in this study. Download Table S3, DOCX file, 0.01 MB. Copyright © 2021 Dorado-Morales et al. 2021 Dorado-Morales et al. https://creativecommons.org/licenses/by/4.0/ This content is distributed under the terms of the Creative Commons Attribution 4.0 International license.

### DNA manipulations.

Routine DNA manipulations were performed using standard procedures unless otherwise indicated. Oligonucleotides were synthesized by StabVida (Caparica, Portugal). FastDigest restriction enzymes, Phusion DNA polymerase, and a rapid DNA ligation kit (Thermo Scientific) were used according to the manufacturer’s instructions. Plasmids were purified using a Macherey-Nagel plasmid purification kit according to the manufacturer’s protocol. To extract plasmids from S. aureus, an extra step was included in the established protocol: after resuspension in buffer A1 and prior to the addition of A2, the mixture was subjected to physical lysis using glass beads (FastPrep cell disrupter; 6 m s^−1^, 40 s). Plasmids were transformed in Escherichia coli by electroporation (1-mm cuvette; 200 Ω, 25 μF, 1,250 V; Gene Pulser X-Cell electroporator). S. aureus competent cells were generated as previously described (35). All plasmids but pLAC-p03 were individually transformed in Staphylococcus aureus wild-type and cured strains by electroporation (1-mm cuvette; 100 Ω, 25 μF, 1,250 V; Gene Pulser X-Cell electroporator). All constructed plasmids were confirmed by Sanger sequencing.

Genomic DNA was extracted from 1 ml of overnight cultures grown in antibiotic-free TSB medium using a GenElute bacterial genomic DNA kit (Sigma) according to the manufacturer’s protocol.

### Moving pLAC-p03 to RN4220.

Plasmids extracted from S. aureus strain LAC(pLAC-p01 pLAC-p03) were transformed into the laboratory strain RN4220, generating an RN4220 strain containing both plasmids. 80α phage lysate was generated as specified in reference 36. A culture of RN4220(pLAC-p01 pLAC-p03) was infected with the 80α lysate at a multiplicity of infection of 0.1 until complete lysis was reached. RN4220(pLAC-p01 pLAC-p03) lysate was filtered (0.2 μm) and used to infect RN4220 wild-type cells. Transductant selection was made in medium containing 100 mM sodium citrate and 0.05 mM cadmium as a selective agent. The presence of pLAC-p03 in Cd-resistant transductants was checked by plasmid extraction followed by PCR amplification using oligonucleotides 365 and 366. The absence of pLAC-p01 was confirmed using primers 280 and 282.

### Construction of pEMPTY::sgRNA2.

The shuttle vector pEMPTY was constructed as follows (Fig. S1). The *cas9* gene and the transcriptional terminator for bacteriophage T7 RNA polymerase were PCR amplified from pFREE-Amp using primers 191 and 473 (25). The tetracycline-inducible promoter P*_xyl_*_/_*_tetO_* was amplified using the pRMC2 plasmid as a template and oligonucleotides 119 and 195 (37). A pCN38 backbone was amplified using oligonucleotides 474 and 189 (38). Then, In-Fusion cloning (In-Fusion HD cloning kit; TaKaRa) was used to combine the three fragments described above. The resulting plasmid was linearized with ApaI and EheI and ligated to the temperature-sensitive origin of replication pE194_ts_-ori amplified from pMAD (39) using oligonucleotides 257 and 259, generating the thermosensitive plasmid pEMPTY.

The module containing the strong constitutive promoter SP01 directing the expression levels of the chimeric molecule composed of guide RNA 2 fused to the transactivating RNA (tracrRNA) was amplified from pFREE-Amp (25) using oligonucleotides 448 and 475. The amplification product was cloned into pJET1.2, digested with SmaI and AscI, gel purified, and cloned into pEMPTY, generating pEMPTY::sgRNA2.

### sgRNA site identification and database generation.

The sequences of 358 fully sequenced staphylococcal plasmids were retrieved from the RefSeq database (40) (release 83). From those plasmids, sequences corresponding to origins of replication and origins of transfer (*oriT*s) were identified through conventional alignments using BLASTn against previously annotated *ori* sequences in the RefSeq files (NC_008356 [pLNU plasmids], NC_001995 [pSK plasmids], NC_001393 [pT181], NC_006974 [pACK6], NC_005566 [pSK639], NC_005243 [pC223], and NC_005127 [pUB101]) and previously identified *oriT*s (41). Sequences corresponding to *rep* and *mob* genes were extracted using HMMER3 (42) and MOBscan software (43). Specific plasmid-related sequences previously described in the literature (44) were also considered.

The online platform sgRNA scorer 2.0 (16) was then used to predict and identify sgRNA activity on the previous plasmid-related sequence database. A score greater than or equal to 0.5, according to the platform classifier, and virtual capability of action over 10 or more plasmids were the requirements established for a target site to be selected. This information can be found in Data Set S1 in the supplemental material.

### Plasmid curing from S. aureus.

S. aureus strains to be cured were transformed with 1 μg of pEMPTY::sgRNA2 plasmid. Recombinant bacteria were selected on solid medium supplemented with 20 μg ml^−1^ Cm after 48 to 72 h of incubation at 28°C, and pEMPTY::sgRNA2 presence was confirmed by PCR using primers 217 and 235. Positive clones were propagated in 5 ml of TSB containing 20 μg ml^−1^ Cm for 8 h (200 rpm, 28°C). Portions (100 μl) of these cultures were used to inoculate 15 ml of fresh TSB medium supplemented with 20 μg ml^−1^ Cm and 100 ng ml^−1^ anhydrotetracycline. After incubation for 16 h at 200 rpm and 28°C, serial dilutions of the cultures were plated on antibiotic-free TSA medium and on TSA containing a selective agent (Cd, Ery, or Lin) to which plasmid-located genes are known to confer resistance. The presence/absence of natural plasmids was doubled checked in colonies isolated on the general medium by streaking on general and selective medium and PCR amplification. The presence of plasmids pMW2, pLAC-p03, pN315, pUR2940, pUR1902, and pUR3912 was checked using primers 365 and 366; the presence of pUR1841 was checked using primers 253 and 255; the presence of pUR2355 was checked using primers 437 and 438.

To remove pEMPTY::sgRNA2, and since it carries the temperature-sensitive origin of replication pE194_ts_-ori, a natural plasmid-free colony was grown in 15 ml of nonselective TSB medium for 16 h at 37°C with shaking. Serial dilutions of the culture were made and plated on antibiotic-free TSA medium and medium containing 20 μg ml^−1^ Cm. Colonies obtained on antibiotic-free TSA were doubled checked for the absence of pEMPTY::sgRNA2 by streaking on general and selective medium and PCR amplification (primers 217 and 235).

### Fitness evaluation.

Overnight cultures of plasmid-free and plasmid-carrying strains were diluted to an optical density at 595 nm (OD_595_) of 0.1 in fresh TSB medium. Portions (5 μl) of the adjusted cultures were used to inoculate 195 μl of fresh TSB medium (10 replicates of each) using 96-well plates (Thermo Scientific). Growth kinetics were assayed using a Synergy H1 hybrid multimode microplate reader (Biotek). Nonselective TSB medium was used in all cases. Growth data (OD_595_) were collected every 15 min for 24 h at 37°C with shaking (fast orbital shaking; 425 cpm). All the experiments were repeated at least three times, and results from one representative experiment are shown.

Two parameters were extracted from growth dynamics: the area under the growth curve (culture total growth) and the duration of the lag phase (45). The mathematical procedure to calculate the area under the growth curve consisted of integrating the OD_595_ values generated from time zero to 6.5 h for each culture. To determine the length of the lag phase, the maximum slope of the growth curve based on 5 consecutive time points was considered the start of the exponential phase of growth.

### Evolution assays.

Three plasmid-carrying clones selected from three independent transformation rounds of the parental plasmid-free strain MW2 PF_t0_ with plasmids pMW2_t0_, pUR2940_t0_, pN315_t0_, and pLAC-p03_t0_ were propagated in 3 ml of antibiotic-free TSB medium, and every 12 h, 3 μl of each culture was transferred to 3 ml of fresh TSB medium for a total duration of 35 days. At the end of the evolution process, cultures were plated on TSA medium containing 0.05 mM cadmium, and a resistant colony was selected for each culture.

Evolution of strain MW2 PF_t0_ transformed with evolved pUR2940_t35_ plasmids and strain C2940 was performed as described above but for a period of 14 days.

### Monitoring of plasmid-carrying cells during the evolution assays.

Every 7 days, evolving cultures were serially diluted and plated on TSA and on TSA containing 0.05 mM Cd or 10 μg ml^−1^ Ery in the case of the C2940 strain. According to the number of resistant colonies, the percentage of plasmid-carrying colonies was calculated. Also, PCR amplification (primers 365 and 366) was randomly performed to discard the emergence of spontaneous mutants on TSA supplemented with Cd and ensure plasmid presence.

### Quantification of plasmid copy number.

Plasmid copy number was determined by quantitative PCR (qPCR) using a QuantStudio 12K Flex real-time PCR system (Life Technologies) and Power SYBR green PCR master mix (Applied Biosystems) at a final DNA concentration of 0.2 ng μl^−1^. For that, DNA extraction was performed with the GenElute bacterial genomic DNA kit (Sigma), DNA was quantified, and 1 μg of genomic DNA was digested with 1 μl of FastDigest EcoRI for 2 h at 37°C. EcoRI was inactivated at 80°C for 15 min. qPCR was performed for each extraction in triplicate. The amplification conditions were initial denaturation for 10 min at 95°C, followed by 40 cycles of denaturation for 15 s at 95°C, annealing (Table S3, qPCR), and extension for 1 min at 57°C. After the amplification was complete, a melting curve analysis was performed by cooling the reaction mixture to 60°C and then heating slowly to 95°C. Primers used for analysis are described in Table S3 (qPCR). The number of plasmid copies per chromosome was calculated (7) as [(1 + *E*_c_)*^CT^*^c^/(1 + *E*_p_)*^CT^*^p^] × (*S*_c_/*S*_p_), where *E*_c_ and *E*_p_ are the efficiencies of the chromosomal and plasmid qPCRs (relative to 1), *CT*c and *CT*p are the threshold cycles of the chromosomal and plasmid reactions, and *S*_c_ and *S*_p_ are the sizes of the chromosomal and plasmid amplicons (in base pairs).

To calculate primer efficiency, serial dilutions of the MW2 wild type EcoRI-digested genomic DNA were used to create a standard curve for each pair of primers. The first point of the curve was dilution 10^−2^. Three technical replicates were made for each point. The average cycle threshold (*C_T_*) was obtained for each point and plotted against the log_10_ of each sample dilution. The slope of the regression was obtained, and the efficiency of the primers was calculated using the following formula (8): (10^1/slope^ − 1) × 100.

### Next-generation sequencing and data processing.

Deep sequencing of DNA samples was performed by MicrobesNG (Birmingham, United Kingdom) using Illumina technology. Genome assembly and plasmid reconstruction were performed with PLACNETw (46, 47). Short-read sequence data were mapped to reference strain S. aureus MW2 (NC_003923.1) or reference plasmid pUR2940 (HF583292.1) by using Snippy (v4.3.6) (https://github.com/tseemann/snippy). Genomes and variant calling files were visualized using IGV (48). BLAST Ring Image Generator (BRIG) was used for comparing plasmid variants (49).

### Statistics.

Statistical analyses were performed with the GraphPad Prism 5.01 program. A one-way analysis of variance combined with the Bonferroni multiple *post hoc* test was used to analyze statistical significance in the study of the initial fitness cost after MW2 PF_t0_ transformation with different plasmids. A nonparametric Mann-Whitney U test was used to assess differences in the analysis of plasmid copy number and fitness cost. Differences with a *P* value of <0.05 were considered significant. Box plots show medians (horizontal black lines), lower and upper quartiles, and extreme values (whiskers).

### Data availability.

The nucleotide sequence of plasmid pEMPTY::sgRNA2 has been deposited in the GenBank database under accession number MW367468. The nucleotide sequences of plasmid pUR2940 and its evolved fit variant determined in this study have been deposited in the GenBank database under accession numbers MW367469 and MW367470, respectively.
