# Supplementary figures and images for: Fitness Cost Evolution of Natural Plasmids of Staphylococcus aureus
Source: mBio. 2021 Feb 23;12(1):e03094-20. doi: 10.1128/mBio.03094-20 (PMC8545097; doi:10.1128/mBio.03094-20)

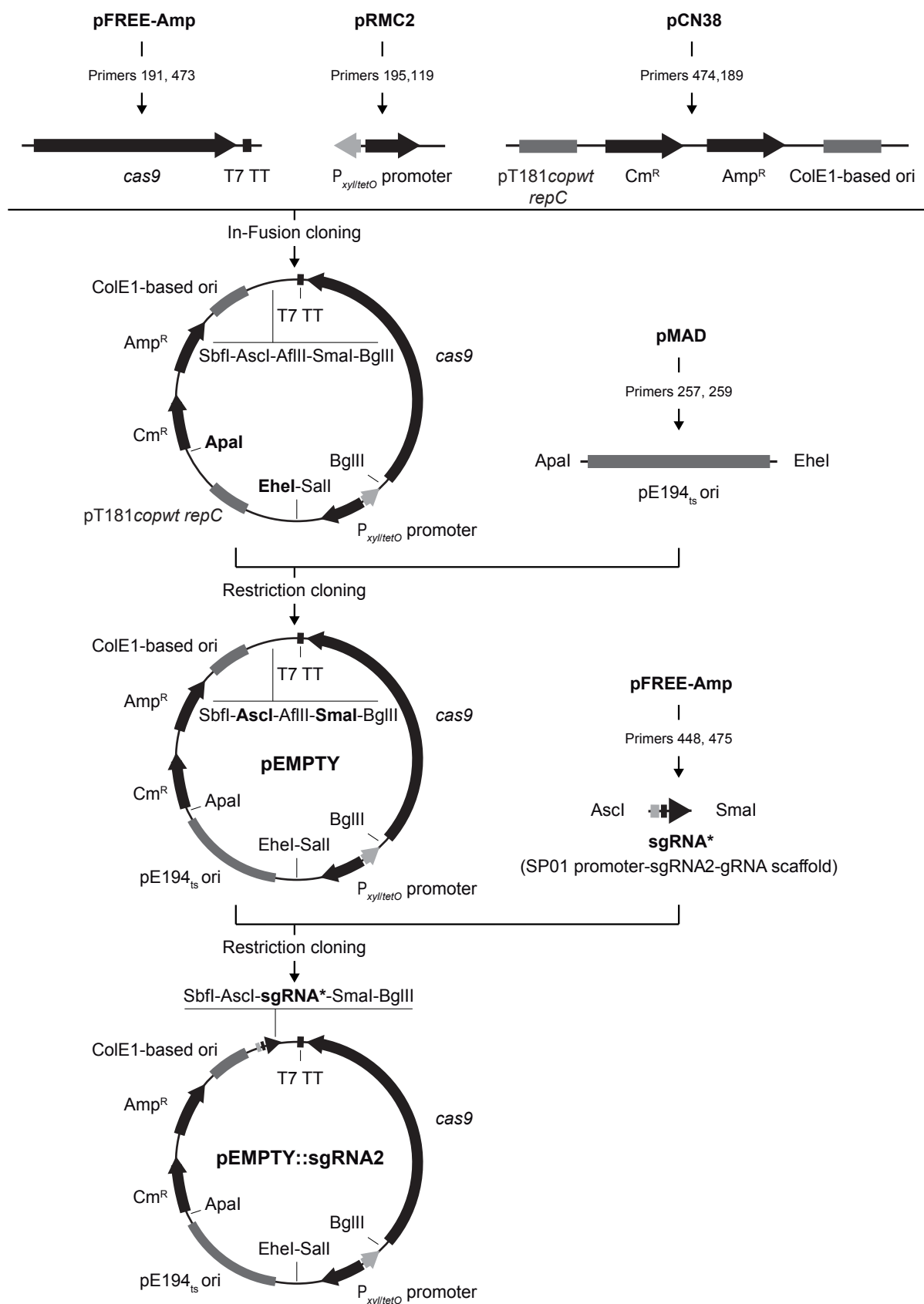

**Fig. S1. Schematic representation of the construction of the pEMPTY::sgRNA2 plasmid.**

Supplement: FIG S1 [file mbio.03094-20-sf001.pdf]
