# Supplementary material for: Fitness Cost Evolution of Natural Plasmids of Staphylococcus aureus
Source: mBio. 2021 Feb 23;12(1):e03094-20. doi: 10.1128/mBio.03094-20 (PMC8545097; doi:10.1128/mBio.03094-20)
Supplement: TABLE S1 [file mbio.03094-20-st001.docx]

**Table S1.** Strains used in this study

| **Strains** | **Relevant characteristics** | **MIC^a^** | **Reference** |
| --- | --- | --- | --- |
| ***Staphylococcus aureus*** | | | |
| RN10359 | RN450 lysogenic for 80α phage | 3337 | (1) |
| RN4220 | Restriction deficient derivative of 8325-4 | 99 | (2) |
| MW2 | Community-acquired methicillin-resistant *Staphylococcus aureus* (CA-MRSA). Clonal lineage USA400. Clonal complex CC1. It contains pMW2 | 3566 | (3) |
| LAC | Community-acquired methicillin-resistant *Staphylococcus aureus* (CA-MRSA). Clonal lineage USA300. Clonal complex CC8. It contains pLAC-p03 and pLAC-p01 | 3413 | (4, 5) |
| N315 | Hospital-acquired methicillin-resistant *Staphylococcus aureus* (HA-MRSA). Clonal lineage USA100. Clonal complex CC5. It contains pN315 | 686 | (6) |
| C2940 | Livestock-associated methicillin-resistant *Staphylococcus aureus* (LA-MRSA). Sequence type ST398. Clonal complex CC398. It contains pUR2940 | 6706 | (7) |
| C1902 | Livestock-associated methicillin-resistant *Staphylococcus aureus* (LA-MRSA). Sequence type ST398. Clonal complex CC398. It contains pUR1902 | 6704 | (7) |
| C3912 | Livestock-associated methicillin-susceptible *Staphylococcus aureus* (LA-MSSA). Sequence type ST398. Clonal complex CC398. It contains pUR3912 as an extrachromosomal element as well as integrated into the chromosome | 6710 | (8, 9) |
| C2355 | Livestock-associated methicillin-resistant *Staphylococcus aureus* (LA-MRSA). Sequence type ST398. Clonal complex CC398. It contains pUR2355 | 6712 | (10) |
| C1841 | Livestock-associated methicillin-resistant *Staphylococcus aureus* (LA-MRSA). Sequence type ST398. Clonal complex CC398. It contains pUR1841 | 6714 | (11) |
| RN4220 pMW2 | RN4220 transformed with pMW2 | 7066 | This study |
| RN4220 pLAC-p03 | RN4220 transduced with pLAC-p03 | 7134 | This study |
| RN4220 pN315 | RN4220 transformed with pN315 | 7065 | This study |
| C4864 | RN4220 transformed with pUR2940 | 6707 | (7) |
| C4858 | RN4220 transformed with pUR1902 | 6705 | (7) |
| C4863 | RN4220 transformed with pUR3912 | 6711 | (9) |
| C4043 | RN4220 pUR2355. RN4220 strain transformed with plasmid pUR2355 | 6713 | (10) |
| C4044 | RN4220 transformed with pUR1841 | 6715 | (11) |
| MW2 PF_t0_ | MW2 cured of plasmid pMW2 | 7068 | This study |
| LAC PF_t0_ | LAC cured of plasmid pLAC-p01 and pLAC-p03 | 7131 | This study |
| LAC PF_t0_ pLAC-p03 | LAC PF_t0_ transformed with pLAC-p03 | 7177 | This study |
| N315 PF_t0_ | N315 cured of plasmid pN315 | 7060 | This study |
| C2940 PF_t0_ | C2940 cured of plasmid pUR2940 | 7058 | This study |
| C1902 PF_t0_ | C1902 cured of plasmid pUR1902 | 7057 | This study |
| C1841 PF_t0_ | C1841 cured of plasmid pUR1841 | 7059 | This study |
| PF_t0_ pMW2_t0_ C1 | MW2 PF_t0_ transformed with plasmid pMW2. Clone 1 | 7090 | This study |
| PF_t0_ pMW2_t0_ C2 | MW2 PF_t0_ transformed with plasmid pMW2. Clone 2 | 7138 | This study |
| PF_t0_ pMW2_t0_ C3 | MW2 PF_t0_ transformed with plasmid pMW2. Clone 3 | 7139 | This study |
| PF_t0_ pUR2940_t0_ C1 | MW2 PF_t0_ transformed with plasmid pUR2940. Clone 1 | 7092 | This study |
| PF_t0_ pUR2940_t0_ C2 | MW2 PF_t0_ transformed with plasmid pUR2940. Clone 2 | 7140 | This study |
| PF_t0_ pUR2940_t0_ C3 | MW2 PF_t0_ transformed with plasmid pUR2940. Clone 3 | 7141 | This study |
| PF_t0_ pN315_t0_ C1 | MW2 PF_t0_ transformed with plasmid pN315. Clone 1 | 7091 | This study |
| PF_t0_ pN315_t0_ C2 | MW2 PF_t0_ transformed with plasmid pN315. Clone 2 | 7132 | This study |
| PF_t0_ pN315_t0_ C3 | MW2 PF_t0_ transformed with plasmid pN315. Clone 3 | 7133 | This study |
| PF_t0_ pLAC-p03_t0_ C1 | MW2 PF_t0_ transformed with plasmid pLAC-p03. Clone 1 | 7135 | This study |
| PF_t0_ pLAC-p03_t0_ C2 | MW2 PF_t0_ transformed with plasmid pLAC-p03. Clone 2 | 7136 | This study |
| PF_t0_ pLAC-p03_t0_ C3 | MW2 PF_t0_ transformed with plasmid pLAC-p03. Clone 3 | 7137 | This study |
| PF_t35_ pMW2_t35_ C1 | PF_t0_ pMW2_t0_ C1 evolved for 35 days under laboratory conditions | EVO10 | This study |
| PF_t35_ pMW2_t35_ C2 | PF_t0_ pMW2_t0_ C2 evolved for 35 days under laboratory conditions | EVO13 | This study |
| PF_t35_ pMW2_t35_ C3 | PF_t0_ pMW2_t0_ C3 evolved for 35 days under laboratory conditions | EVO16 | This study |
| PF_t35_ pUR2940_t35_ C1 | PF_t0_ pUR2940_t0_ C1 evolved for 35 days under laboratory conditions | EVO19 | This study |
| PF_t35_ pUR2940_t35_ C2 | PF_t0_ pUR2940_t0_ C2 evolved for 35 days under laboratory conditions | EVO22 | This study |
| PF_t35_ pUR2940_t35_ C3 | PF_t0_ pUR2940_t0_ C3 evolved for 35 days under laboratory conditions. | EVO25 | This study |
| PF_t35_ pN315_t35_ C1 | PF_t0_ pN315_t0_ C1 evolved for 35 days under laboratory conditions | EVO28 | This study |
| PF_t35_ pN315_t35_ C2 | PF_t0_ pN315_t0_ C2 evolved for 35 days under laboratory conditions | EVO31 | This study |
| PF_t35_ pN315_t35_ C3 | PF_t0_ pN315_t0_ C3 evolved for 35 days under laboratory conditions | EVO34 | This study |
| PF_t35_ pLAC-p03_t35_ C1 | PF_t0_ pLAC-p03_t0_ C1 evolved for 35 days under laboratory conditions | EVO37 | This study |
| PF_t35_ pLAC-p03_t35_ C2 | PF_t0_ pLAC-p03_t0_ C2 evolved for 35 days under laboratory conditions | EVO40 | This study |
| PF_t35_ pLAC-p03_t35_ C3 | PF_t0_ pLAC-p03_t0_ C3 evolved for 35 days under laboratory conditions | EVO43 | This study |
| PF_t35_ C1 | PF_t35_ pUR2940_t35_ C1 cured of plasmid pUR2940 | EVO62 | This study |
| PF_t35_ C2 | PF_t35_ pUR2940_t35_ C2 cured of plasmid pUR2940 | EVO63 | This study |
| PF_t35_ C3 | PF_t35_ pUR2940_t35_ C3 cured of plasmid pUR2940 | EVO64 | This study |
| PF_t35_ C1 pUR2940_t0_ | PF_t35_ C1 transformed with plasmid isolated from PF_t0_ pUR2940_t0_ C1 | EVO74 | This study |
| PF_t35_ C2 pUR2940_t0_ | PF_t35_ C2 transformed with plasmid isolated from PF_t0_ pUR2940_t0_ C2 | EVO75 | This study |
| PF_t35_ C3 pUR2940_t0_ | PF_t35_ C3 transformed with plasmid isolated from PF_t0_ pUR2940_t0_ C3 | EVO76 | This study |
| PF_t0_ pUR2940_t35_ C1 | MW2 PF_t0_ transformed with plasmid isolated from PF_t35_ pUR2940_t35_ C1 | EVO50 | This study |
| PF_t0_ pUR2940_t35_ C2 | MW2 PF_t0_ transformed with plasmid isolated from PF_t35_ pUR2940_t35_ C2 | EVO51 | This study |
| PF_t0_ pUR2940_t35_ C3 | MW2 PF_t0_ transformed with plasmid isolated from PF_t35_ pUR2940_t35_ C3 | EVO52 | This study |
| ***Escherichia coli*** |  |  |  |
| IMO1B | *mcrA* Δ(*mrr*-*hsdRMS*-*mcrBC*) φ80*lacZ*ΔM15 Δ*lacX*74 *recA*1 *araD*139 Δ(*ara*-*leu*)7697 *galU* *galK* *rpsL* *endA*1 *nupG* Δ*dcm* Ω*Phelp*-*hsdMS* (CC1-2) ΩPN25-*hsdS* (CC1-1).  *E. coli* K12 DH10B derivative. Δ*dcm* (gene encoding cytosine methylation). The *hsdMS* genes encoding methylase and specificity genes from *Staphylococcus aureus* MW2 clonal complex 1 were introduced into the chromosome at neutral locations via recombineering. | 5694 | (12) |

^a^ Number of each strain in the culture collection of the Laboratory of Microbial Pathogenesis, Navarrabiomed-Universidad Pública de Navarra.

**References**

1. Ubeda C, Barry P, Penadés JR, Novick RP. 2007. A pathogenicity island replicon in *Staphylococcus aureus* replicates as an unstable plasmid. Proc Natl Acad Sci USA 104:14182–14188.

2. Peng HL, Novick RP, Kreiswirth B, Kornblum J, Schlievert P. 1988. Cloning, characterization, and sequencing of an accessory gene regulator (*agr*) in *Staphylococcus aureus*. J Bacteriol 170:4365–4372.

3. Baba T, Takeuchi F, Kuroda M, Yuzawa H, Aoki K, Oguchi A, Nagai Y, Iwama N, Asano K, Naimi T, Kuroda H, Cui L, Yamamoto K, Hiramatsu K. 2002. Genome and virulence determinants of high virulence community-acquired MRSA. Lancet 359:1819–1827.

4. Kennedy AD, Otto M, Braughton KR, Whitney AR, Chen L, Mathema B, Mediavilla JR, Byrne KA, Parkins LD, Tenover FC, Kreiswirth BN, Musser JM, DeLeo FR. 2008. Epidemic community-associated methicillin-resistant *Staphylococcus aureus*: Recent clonal expansion and diversification. Proc Natl Acad Sci USA 105:1327–1332.

5. Kennedy AD, Porcella SF, Martens C, Whitney AR, Braughton KR, Chen L, Craig CT, Tenover FC, Kreiswirth BN, Musser JM, DeLeo FR. 2010. Complete nucleotide sequence analysis of plasmids in strains of *Staphylococcus aureus* clone USA300 reveals a high level of identity among isolates with closely related core genome sequences. J Clin Microbiol 48:4504–4511.

6. Kuroda M, Ohta T, Uchiyama I, Baba T, Yuzawa H, Kobayashi I, Cui LZ, Oguchi A, Aoki K, Nagai Y, Lian JQ, Ito T, Kanamori M, Matsumaru H, Maruyama A, Murakami H, Hosoyama A, Mizutani-Ui Y, Takahashi NK, Sawano T, Inoue R, Kaito C, Sekimizu K, Hirakawa H, Kuhara S, Goto S, Yabuzaki J, Kanehisa M, Yamashita A, Oshima K, Furuya K, Yoshino C, Shiba T, Hattori M, Ogasawara N, Hayashi H, Hiramatsu K. 2001. Whole genome sequencing of meticillin-resistant *Staphylococcus aureus*. Lancet 357:1225–1240.

7. Gómez-Sanz E, Kadlec K, Feßler AT, Zarazaga M, Torres C, Schwarz S. 2013. Novel *erm*(T)-carrying multiresistance plasmids from porcine and human isolates of methicillin-resistant *Staphylococcus aureus* ST398 that also harbor cadmium and copper resistance determinants. Antimicrob Agents Chemother 57:3275–3282.

8. Gómez-Sanz E, Kadlec K, Feßler AT, Billerbeck C, Zarazaga M, Schwarz S, Torres C. 2013. Analysis of a novel *erm*(T)- and *cadDX*-carrying plasmid from methicillin-susceptible *Staphylococcus aureus* ST398-t571 of human origin. J Antimicrob Chemother 68:471–473.

9. Gómez-Sanz E, Zarazaga M, Kadlec K, Schwarz S, Torres C. 2013. Chromosomal integration of the novel plasmid pUR3912 from methicillin-susceptible *Staphylococcus aureus* ST398 of human origin. Clin Microbiol Infect 19:E519–22.

10. Lozano C, Aspiroz C, Rezusta A, Gómez-Sanz E, Simon C, Gómez P, Ortega C, Revillo MJ, Zarazaga M, Torres C. 2012. Identification of novel *vga*(A)-carrying plasmids and a Tn5406-like transposon in meticillin-resistant *Staphylococcus aureus* and *Staphylococcus epidermidis* of human and animal origin. Int J Antimicrob Agents 40:306–312.

11. Lozano C, Aspiroz C, Sáenz Y, Ruiz-García M, Royo-García G, Gómez-Sanz E, Ruiz-larrea F, Zarazaga M, Torres C. 2012. Genetic environment and location of the *lnu*(A) and *lnu*(B) genes in methicillin-resistant *Staphylococcus aureus* and other staphylococci of animal and human origin. J Antimicrob Chemother 67:2804–2808.

12. Monk IR, Tree JJ, Howden BP, Stinear TP, Foster TJ. 2015. Complete bypass of restriction systems for major *Staphylococcus aureus* lineages. mBio 6:1–12.
