# Supplementary material for: Fitness Cost Evolution of Natural Plasmids of Staphylococcus aureus
Source: mBio. 2021 Feb 23;12(1):e03094-20. doi: 10.1128/mBio.03094-20 (PMC8545097; doi:10.1128/mBio.03094-20)
Supplement: FIG S2 [file mbio.03094-20-sf002.pdf]

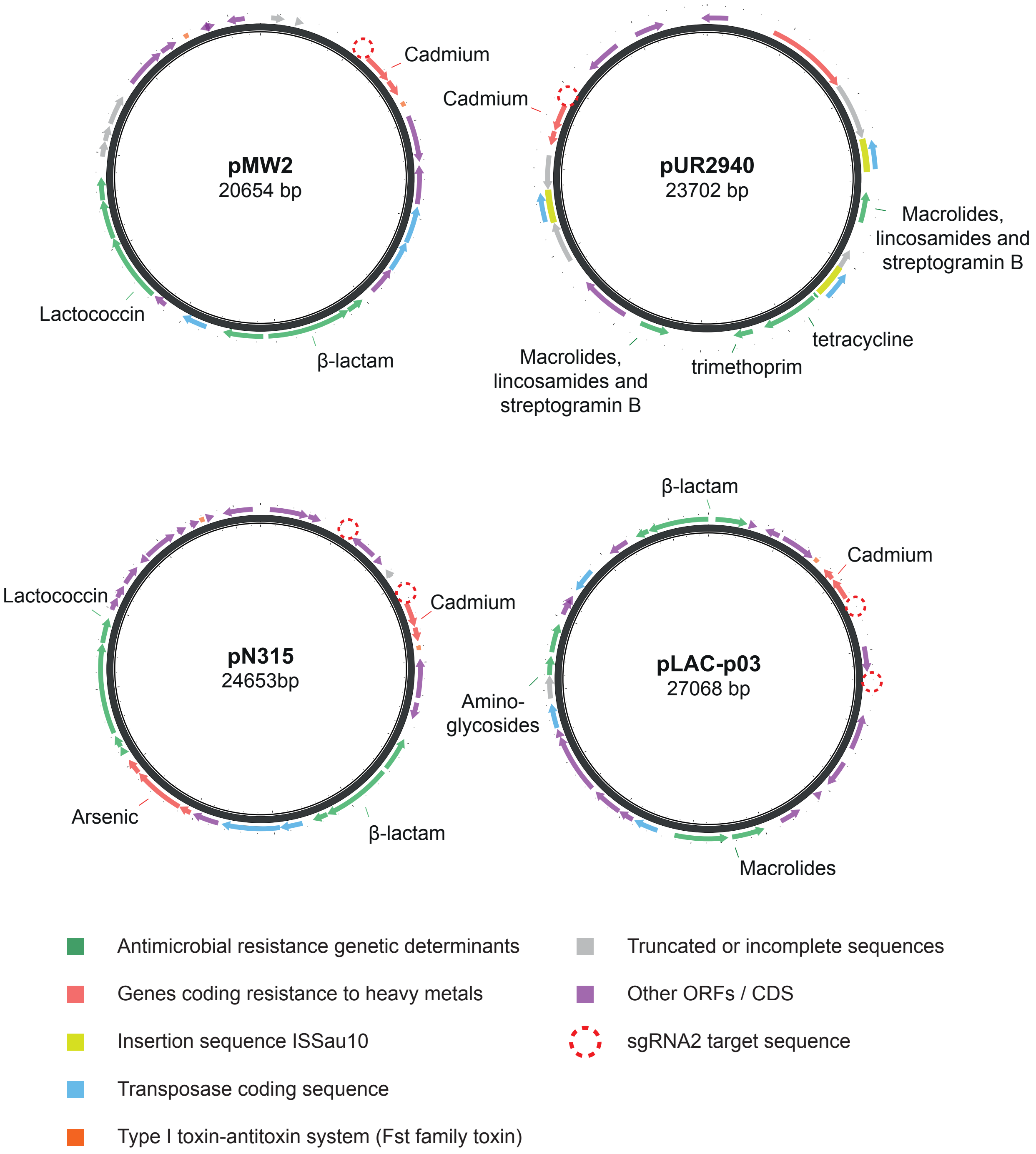

Fig. S2. Maps of plasmids pMW2, pUR2940, pN315 and pLAC-p03. Images were generated with the BLAST Ring Image Generator (BRIG) using plasmid sequences retrieved from the NCBI RefSeq database or obtained by Illumina sequencing.
