# Supplementary material for: Fitness Cost Evolution of Natural Plasmids of Staphylococcus aureus
Source: mBio. 2021 Feb 23;12(1):e03094-20. doi: 10.1128/mBio.03094-20 (PMC8545097; doi:10.1128/mBio.03094-20)
Supplement: FIG S3 [file mbio.03094-20-sf003.pdf]

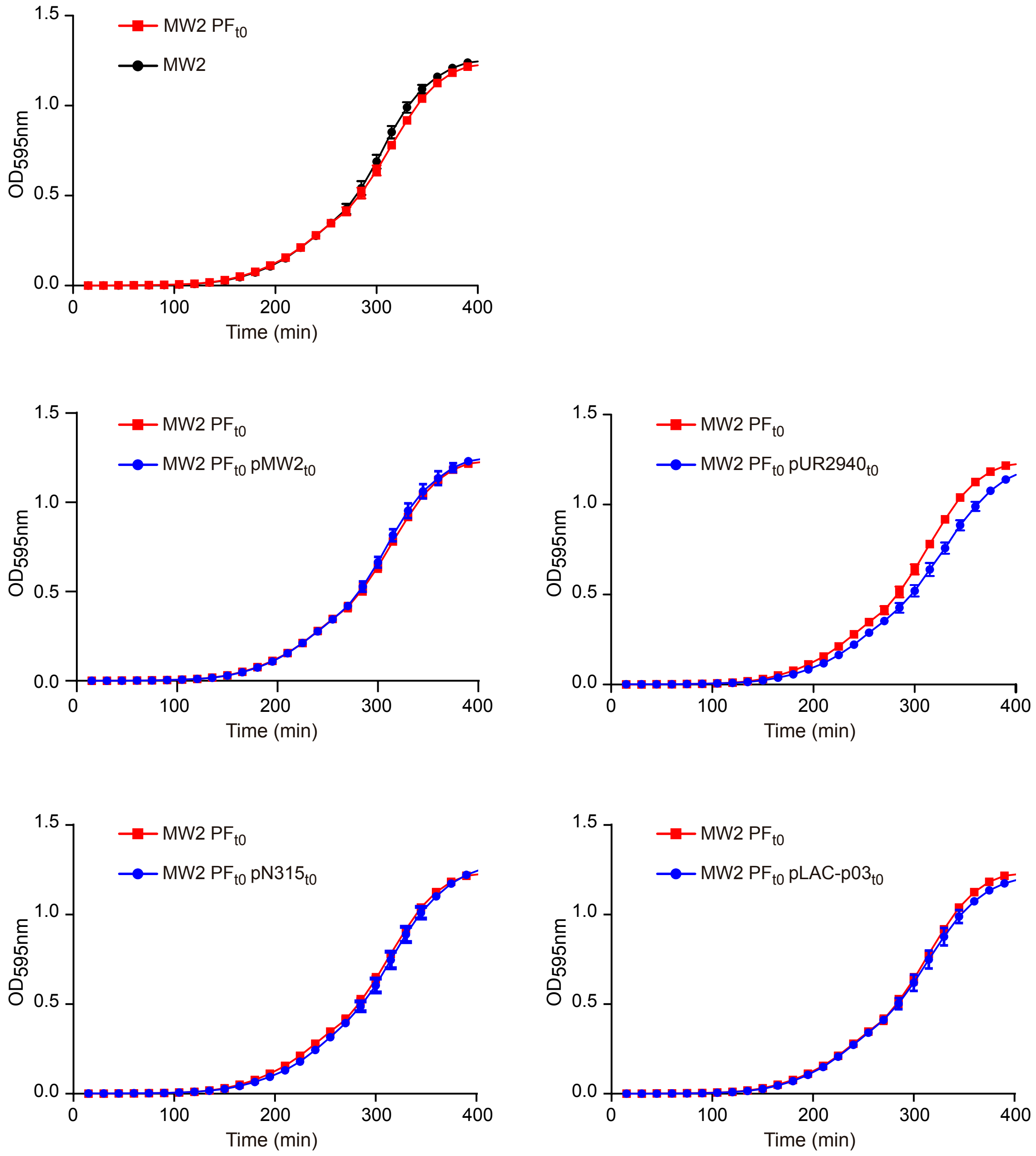

Fig. S3. Growth curve of plasmid-free MW2 (MW2 PF_{t0}) compared with that of the wild type MW2 strain and MW2 PF_{t0} transformed with plasmids pMW2, pUR2940, pN315, and pLAC-p03. Data were collected every 15 minutes during growth in TSB medium in 96 multi-well plates for 6.5 hours at 37 ˚C under shaking conditions. The average OD_{595nm} values and standard deviation from ten technical replicates were plotted for each of the tested strains. In the case of MW2 PF_{t0} transformed with plasmids pMW2, pUR2940, pN315, and pLAC-p03, results of one representative clone of the three independently transformed clones are shown.
