# Supplementary material for: Fitness Cost Evolution of Natural Plasmids of Staphylococcus aureus
Source: mBio. 2021 Feb 23;12(1):e03094-20. doi: 10.1128/mBio.03094-20 (PMC8545097; doi:10.1128/mBio.03094-20)
Supplement: FIG S4 [file mbio.03094-20-sf004.pdf]

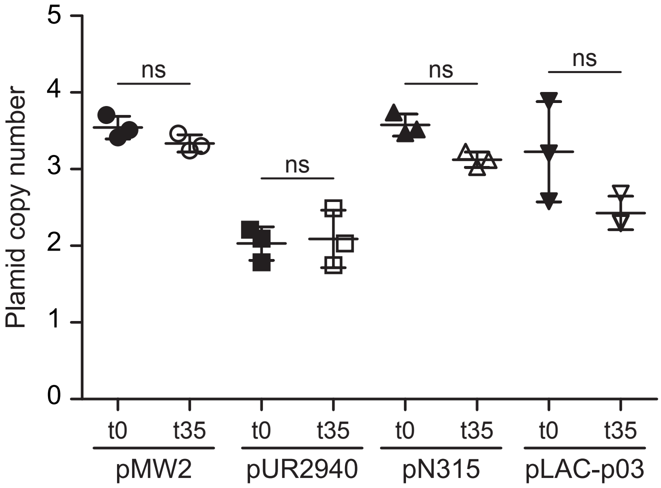

Fig. S4. Plasmid copy number at days 0 and day 35 of the evolution experiment. Number of pMW2, pUR2940, pN315 and pLAC-p03 copies per cell in the three ancestral MW2 transformed clones (t0) and the three MW2 plasmid-carrying evolved clones (t35) were determined by qPCR. Data represent the mean and standard deviation of values obtained from the three individual clones analyzed in each case. Experiments were carried out in triplicates. Statistical analysis was carried out using a Mann-Whitney U test. ns; no significant difference.
