# Supplementary material for: Fitness Cost Evolution of Natural Plasmids of Staphylococcus aureus
Source: mBio. 2021 Feb 23;12(1):e03094-20. doi: 10.1128/mBio.03094-20 (PMC8545097; doi:10.1128/mBio.03094-20)
Supplement: TABLE S2 [file mbio.03094-20-st002.docx]

**Table S2.** Plasmids used in this study

| **Plasmids** | **Relevant characteristics** | **Size (pb)** | **Reference** |
| --- | --- | --- | --- |
| pJET1.2 | Cloning vector. Amp^R^ | 2974 | Thermo Scientific |
| pFREE-Amp | Plasmid designed to remove plasmids from *E. coli*. CRISPR-Cas9-based curing. Amp^R^ | 7804 | (1) |
| pRMC2 | *E. coli* - *S. aureus* shuttle vector. Expression of genes from the anhydrotetracycline-inducible P*_xyl_*_/_*_tetO_* promoter. Amp^R^ Cm^R^ | 6555 | (2) |
| pCN38 | *E. coli* - *S. aureus* shuttle vector. Amp^R^ Cm^R^ | 5426 | (3) |
| pMAD | *E. coli* - *S. aureus* shuttle vector containing a thermosensitive origin of replication for Gram-positive bacteria. Amp^R^ Ery^R^ | 9666 | (4) |
| pEMPTY | *E. coli* - *S. aureus* shuttle vector with the *cas9* gene expression under the P*_xyl_*_/_*_tetO_* promoter. Thermosensitive origin of replication for Gram-positive bacteria. Amp^R^ Cm^R^ | 10497 | This study |
| pEMPTY::sgRNA2 | *E. coli* - *S. aureus* shuttle vector that allows CRISPR-Cas9-based rapid and efficient plasmid curing in *Staphylococcus aureus*. *cas9* gene expression under the P*_xyl_*_/_*_tetO_* promoter. sgRNA2 expression under SP01 promoter. Thermosensitive origin of replication for Gram-positive bacteria. Amp^R^ Cm^R^ | 11271 | This study |
| pMW2 | Naturally occurring plasmid. Isolated from *S. aureus* MW2 | 20654 | (5) |
| pUR2940 | Naturally occurring plasmid. Isolated from *S. aureus* C2940 | 23702 | (6) |
| pN315 | Naturally occurring plasmid. Isolated from *S. aureus* N315 | 24653 | (7) |
| pLAC-p03 | Naturally occurring plasmid. Isolated from *S. aureus* LAC | 27068 | (8) |
| pUR1902 | Naturally occurring plasmid. Isolated from *S. aureus* C1902 | ≈2000 | (6) |
| pUR3912 | Naturally occurring plasmid. Isolated from *S. aureus* C3912 | 6176 | (9) |
| pUR1841 | Naturally occurring plasmid. Isolated from *S. aureus* C1841 | 2361 | (10) |
| pUR2355 | Naturally occurring plasmid. Isolated from *S. aureus* C2355 | 7609 | (11) |
| pUR2940_t35_ C1 | Plasmid isolated from *S. aureus* PF_t0_ pUR2940_t0_ C1 after being evolved under laboratory conditions for 35 days | 23702 | This study |
| pUR2940_t35_ C2 | Plasmid isolated from *S. aureus* PF_t0_ pUR2940_t0_ C2 after being evolved under laboratory conditions for 35 days | 10822 | This study |
| pUR2940_t35_ C3 | Plasmid isolated from *S. aureus* PF_t0_ pUR2940_t0_ C3 after being evolved under laboratory conditions for 35 days | 12474 | This study |

**References**

1. Lauritsen I, Porse A, Sommer MOA, Nørholm MHH. 2017. A versatile one-step CRISPR-Cas9 based approach to plasmid-curing. Microb Cell Fact 16:1–10.

2. Corrigan RM, Foster TJ. 2009. An improved tetracycline-inducible expression vector for *Staphylococcus aureus*. Plasmid 61:126–129.

3. Charpentier E, Anton AI, Barry P, Alfonso B, Fang Y, Novick RP. 2004. Novel cassette-based shuttle vector system for gram-positive bacteria. Appl Environ Microbiol 70:6076–6085.

4. Arnaud M, Chastanet A, Débarbouillé M. 2004. New vector for efficient allelic replacement in naturally nontransformable, low-GC-content, gram-positive bacteria. Appl Environ Microbiol 70:6887–6891.

5. Baba T, Takeuchi F, Kuroda M, Yuzawa H, Aoki K, Oguchi A, Nagai Y, Iwama N, Asano K, Naimi T, Kuroda H, Cui L, Yamamoto K, Hiramatsu K. 2002. Genome and virulence determinants of high virulence community-acquired MRSA. Lancet 359:1819–1827.

6. Gómez-Sanz E, Kadlec K, Feßler AT, Zarazaga M, Torres C, Schwarz S. 2013. Novel *erm*(T)-carrying multiresistance plasmids from porcine and human isolates of methicillin-resistant *Staphylococcus aureus* ST398 that also harbor cadmium and copper resistance determinants. Antimicrob Agents Chemother 57:3275–3282.

7. Kuroda M, Ohta T, Uchiyama I, Baba T, Yuzawa H, Kobayashi I, Cui LZ, Oguchi A, Aoki K, Nagai Y, Lian JQ, Ito T, Kanamori M, Matsumaru H, Maruyama A, Murakami H, Hosoyama A, Mizutani-Ui Y, Takahashi NK, Sawano T, Inoue R, Kaito C, Sekimizu K, Hirakawa H, Kuhara S, Goto S, Yabuzaki J, Kanehisa M, Yamashita A, Oshima K, Furuya K, Yoshino C, Shiba T, Hattori M, Ogasawara N, Hayashi H, Hiramatsu K. 2001. Whole genome sequencing of meticillin-resistant *Staphylococcus aureus*. Lancet 357:1225–1240.

8. Kennedy AD, Porcella SF, Martens C, Whitney AR, Braughton KR, Chen L, Craig CT, Tenover FC, Kreiswirth BN, Musser JM, DeLeo FR. 2010. Complete nucleotide sequence analysis of plasmids in strains of *Staphylococcus aureus* clone USA300 reveals a high level of identity among isolates with closely related core genome sequences. J Clin Microbiol 48:4504–4511.

9. Gómez-Sanz E, Kadlec K, Feßler AT, Billerbeck C, Zarazaga M, Schwarz S, Torres C. 2013. Analysis of a novel *erm*(T)- and *cadDX*-carrying plasmid from methicillin-susceptible *Staphylococcus aureus* ST398-t571 of human origin. J Antimicrob Chemother 68:471–473.

10. Lozano C, Aspiroz C, Sáenz Y, Ruiz-García M, Royo-García G, Gómez-Sanz E, Ruiz-larrea F, Zarazaga M, Torres C. 2012. Genetic environment and location of the *lnu*(A) and *lnu*(B) genes in methicillin-resistant *Staphylococcus aureus* and other staphylococci of animal and human origin. J Antimicrob Chemother 67:2804–2808.

11. Lozano C, Aspiroz C, Rezusta A, Gómez-Sanz E, Simon C, Gómez P, Ortega C, Revillo MJ, Zarazaga M, Torres C. 2012. Identification of novel *vga*(A)-carrying plasmids and a Tn5406-like transposon in meticillin-resistant *Staphylococcus aureus* and *Staphylococcus epidermidis* of human and animal origin. Int J Antimicrob Agents 40:306–312.
