# Supplementary material for: Fitness Cost Evolution of Natural Plasmids of Staphylococcus aureus
Source: mBio. 2021 Feb 23;12(1):e03094-20. doi: 10.1128/mBio.03094-20 (PMC8545097; doi:10.1128/mBio.03094-20)
Supplement: TABLE S3 [file mbio.03094-20-st003.docx]

**Table S3.** Oligonucleotides used in this study

| **Construction of pEMPTY and pEMPTY::sgRNA2** | | | | | |
| --- | --- | --- | --- | --- | --- |
| Oligonucleotide | Sequence (5’-3’)^a^ | | | | |
| 191 | **aaaaaatattgacactctatcattgatagagtataattaaaataagcttg***AGATCT*TTaggaggatGATTATTTatggataagaaatactcaataggc | | | | |
| 473 | **GAGCTGGCGGCCGCTGCATG*CCTGCAGGGCGCGCCCTTAAGCCCggg*taccAGATCTcaaaaaacccctcaagacccgttt** | | | | |
| 119 | Caagcttattttaattatactctatcaatgatagagtgtcaatattttttttag | | | | |
| 195 | **Gggtaacgccagggttttcccagtcacgacgttgtaaaacgacggccagtgaattc**caggtcgacggtatcgataac | | | | |
| 474 | **aaacgggtcttgaggggttttttgAGATCTggta*cccGGGCTTAAGGGCGCGCCCTGCAG*GCATGCAGCGGCCGCCAGCTC** | | | | |
| 189 | **tttccgtgatggtaacttcacggtaaccaagatgtcgagttatcgataccgtcgacctg**gaattcactggccgtcgtttt | | | | |
| 257 | **GTTGCGCAGCCTGAATGGCGAAT***GGCGCC*TGAGGGTTGCCAGAGTTAAAG | | | | |
| 259 | **CTTTCTTATCTTGATAATACCTAGGT***GGGCCC*CCTCGATCCCGCAAGAGGCC | | | | |
| 448 | *CCCGGGCTTAAG*gttgcgcacaccgactagcg | | | | |
| 475 | *GGCGCGCC*ttgacaaattgcagtaggcatgacaaaatggactcacaagttttgggattgttaagggttccggttttagagctagaaatagcaagttaaaataaggctagtc | | | | |
|  |  | | | | |
| **Checking plasmid presence** | | | | | |
| Oligonucleotide | Sequence (5’-3’) | | | | |
| 217 | cgtaaacggatgctggctag | | | | |
| 235 | cgatttttgtgatgctcgtcaggg | | | | |
| 280 | TGAATGCAATTCAAAACAGTATATCAC | | | | |
| 282 | GTTCTCCATATGAGTTTAAAACTTCAG | | | | |
| 253 | CTGTTAAGTCATAACCAGAATG | | | | |
| 255 | ATGAAAATTAATAATGTAACAGAAAAG | | | | |
| 365 | TGATGTGAAGTTGAAGCAACACTC | | | | |
| 366 | TGATGTGATCTGTGTACATGAGGA | | | | |
| 437 | GGATCATGTACACAACCATA | | | | |
| 438 | TTGTTCTGTGTTGTGTTCGA | | | | |
|  |  | | | | |
| **Unveiling pUR2940_t35_ C2 and C3 final structure** | | | | | |
| Oligonucleotide | Sequence (5’-3’) | | | | |
| 412 | gcatttattggcttgattttatcacctgc | | | | |
| 414 | agggaaacaggttttagtttattcaggtc | | | | |
|  |  | | | | |
| **qPCR** | | | | | |
| Oligonucleotide | Sequence (5’-3’) | Target^b^ | Size^c^ (pb) | Tm^d^ | Primer efficiency |
| 365 | TGATGTGAAGTTGAAGCAACACTC | *cadX* | 207 | 56 | 0.9284 |
| 366 | TGATGTGATCTGTGTACATGAGGA | *cadX* |  |  |  |
| 367 | caataatacgccgcgttaaatctg | *phoR* | 205 | 57 | 0.9388 |
| 368 | tcaattaaggggtttgctgaaacg | *phoR* |  |  |  |
| 369 | TTATGGTGCTGGGCAAATACA | *gyrB* | 338 | 60 | 0.8188 |
| 370 | CACCATGTAAACCACCAGATA | *gyrB* |  |  |  |

^a^ Overlapping sequences for In-Fusion HD cloning, primer annealing regions and restriction enzymes sites are indicated in bold, underlined format and italics, respectively. The ribosome binding site of the superoxide dismutase gene (*sod*) is shown in lower case letters. The SP01 promoter, sgRNA2 and sgRNA scaffold extra sequence are highlighted in light, medium and dark gray, respectively.

^b^ *cadX* (plasmid gene); *phoR* (chromosomal monocopy gene used to compare the ratio of plasmid and chromosomal DNA in all cases except in strain C2940); *gyrB* (chromosomal monocopy gene used to compare the ratio of plasmid and chromosomal DNA in strain C2940).

^c^ Amplicon size.

^d^Melting temperatures used for the qPCR.
